# A worldwide survey on the use of animal‐derived materials and reagents in scientific experimentation

**DOI:** 10.1002/elsc.202100167

**Published:** 2022-07-18

**Authors:** Manuela Cassotta, Joanna Julia Bartnicka, Francesca Pistollato, Surat Parvatam, Tilo Weber, Vito D'Alessandro, Luisa Ferreira Bastos, Sandra Coecke

**Affiliations:** ^1^ Oltre la Sperimentazione Animale (OSA) Segrate Milan Italy; ^2^ European Commission Joint Research Centre (JRC) Ispra Italy; ^3^ Centre for Predictive Human Model Systems Atal Incubation Centre‐Centre for Cellular and Molecular Biology (AIC‐CCMB) Hyderabad India; ^4^ Department for Alternatives to the Use of Animals in Research, Testing and Education Animal Welfare Academy of the German Animal Welfare Federation Neubiberg Germany; ^5^ Eurogroup for Animals Brussels Belgium

**Keywords:** antibodies, FBS, fetal bovine serum, GIVIMP, Guidance Document on Good In vitro Method Practices, in vitro, reproducibility

## Abstract

The use of cell and tissue‐based methods in basic, applied and regulatory science has been increasing exponentially. Animal‐derived components, including serum, coating materials, growth factors and antibodies are routinely used in cell/tissue cultures and in general laboratory practices. In addition to ethical issues, the use and production of animal‐derived materials and reagents raises many scientific concerns, generally associated with presence of undefined components and batch‐to‐batch variability, which may compromise experimental reproducibility. On the other hand, non‐animal materials and reagents, such as human cells, alternatives to animal sera or non‐animal recombinant antibodies, are becoming increasingly available, and their use is encouraged by the EU Directive 2010/63 and the Guidance Document on Good In vitro Method Practices (GIVIMP), published by the Organization for Economic Cooperation and Development (OECD). In an effort to map the current state of use of animal‐derived reagents across different sectors and to identify the obstacles possibly hampering the implementation of non‐animal derived alternatives, a global online survey addressed to scientists working on in vivo, in vitro, in silico methods, in academia as well as pharmaceutical or cosmetic companies, was conducted with the goal to understand: 1) the most commonly used animal‐derived materials and reagents, 2) the main issues associated with the production and use of animal‐derived materials and reagents, 3) the current level of knowledge on available non‐animal alternative materials and reagents, and 4) what educational and information sources could be most useful or impactful to disseminate knowledge on non‐animal alternatives. This paper provides an overview of the survey replies and discusses possible proposals to increase awareness, acceptance and use of non‐animal ingredients.

## 1 INTRODUCTION

Russell and Burch's book *The Principles of Humane Experimental Technique*, published in 1959, first introduced the three principles of Replacement, Reduction and Refinement (3Rs) of animals in scientific experimentation [1]. In line with the principle of Replacement, over the last 60 years, the number of animals used for scientific research in Europe has dropped considerably [2], although the Registration, Evaluation, Authorization and Restriction of Chemicals (REACH)‐program is expected to boost in vivo testing [3, 4]. In accordance with the 3R principles, researchers in different fields have adopted cell and tissue cultures for a variety of objectives, contributing to the reduction and, in some cases, the replacement of animal experiments. The European Union (EU) Directive on the protection of animals used for scientific purposes (Directive 2010/63/EU) is firmly based on the principle of the 3Rs, pursuing the ultimate goal to fully replace the use of animals in basic, translational and applied research, regulatory testing, routine production, as well as education and training. In addition, in recent years, the need for a paradigm shift towards human‐based research has become progressively apparent, as the limitations of animal models are increasingly becoming recognized within several research fields [5, 6, 7]. Consequently, the use of cell and tissue‐based methods in basic, applied, and regulatory science has been increasing exponentially and it is expected to grow further in the coming years.

In line with this, non‐animal materials and reagents, such as human cells and tissues, alternatives to animal sera or non‐animal recombinant antibodies, are becoming increasingly available on the market and from cell/tissue banks.

Fetal bovine serum, also known as fetal calf serum, (FBS/FCS), dissociation enzymes, (e.g., trypsin, papain, etc.), coating materials including collagen, laminin, and Matrigel™ are commonly used for cell culturing, while animal‐derived monoclonal and polyclonal antibodies are being employed for research, therapeutics, diagnostics, and for regulatory purposes. However, there are few regulations relating to the collection of fetal tissues [8]. The World Organisation for Animal Health (*Office International des Epizooties*, OIE) sets guidelines for animal welfare although this mandate does not fall under the World Trade Organization Agreement on the Application of Sanitary and Phytosanitary Measures [9]. In chapter 7.5 of its Terrestrial Code, OIE provides international standards aiming at ensuring that the fetus is dead prior to the tissue collection [10]. However, this is criticized as insufficient to prevent fetal suffering as there is no scientific consensus on whether the fetus is able to suffer while still in utero [8]. Thus, killing of the fetus with a certified killing method (or at least anesthetization) should be performed immediately after the slaughter of the dam [11]. Ideally, it should be ensured that pregnant dams and their fetuses do not end up at slaughterhouses in the first place, an initial step towards that is proposed by Eurogroup for Animals’ white paper on live animal transport: “pregnant animals for whom 40% or more of the expected gestation period has already passed […] shall be considered unfit for transport” [12]. Transparent communication about procedures involving the production and placing on the market of food and feed products, including animal‐derived products, is mandated in the EU Regulation 2019/1381 on the transparency and sustainability of the EU risk assessment in the food chain [13]. At least 25% of the budget for direct payments will be allocated to eco‐schemes, providing stronger incentives for climate‐and environment‐friendly farming practices and approaches (such as organic farming, agro‐ecology, carbon farming, etc.) as well as animal welfare improvements and as such the issue of animal‐derived ingredients drawn from farm animals should be compliant with animal welfare concerns [14].

Rigorous examination of the global use of FBS has shown that the worldwide annual production of FBS is about 600,000 to 800,000 L collected from around 1 to 2 million fetuses [15, 16].

Other animal‐derived materials and reagents, such as Matrigel™ (a basement membrane preparation derived from the Engelbreth‐Holm‐Swarm sarcoma grown and propagated in mice), collagen from diverse animal origins, and animal‐derived antibodies, also results in significant animal welfare concerns, which are in opposition to the original 3Rs principle [17]. Notably, all animal‐derived materials and reagents are obtained from different animal groups and result in batch‐to‐batch variation, which may lead to reliability and integrity concerns for generated in vitro data [18, 19]. In addition, serum is a complex mixture of different factors, which introduces undefined constituents within the culture medium. Many of these components have not yet been identified, and in several cases, their effects on cell/tissue cultures are still unknown [18, 20]. Furthermore, animal‐derived products may also raise contamination issues, for example, due to the presence of viruses [20, 21].

**PRACTICAL APPLICATION**

This survey represents a first effort to map the current state of use of animal‐derived reagents across different sectors and to identify the hurdles possibly hampering the large implementation and use of non‐animal derived alternatives. The conclusions drawn from the survey formed the basis for a series of proposed initiatives. Implementing these initiatives will bring not only ethical but also scientific benefits, considering that there is an urgent scientific need to replace animal‐derived, chemically‐undefined materials and reagents with chemically‐defined, non‐animal alternatives. Biomedical sciences are facing what has been called “the reproducibility crisis”. The crisis is borne out of failures to replicate the results of published research. The extensive use of biologically undefined animal‐derived materials and reagents are proposed as one of the contributors to the reproducibility crisis in biomedical research, while scientificallyrobust non‐animal alternatives are becoming increasingly available. This paper presents a collection of precious information for the scientific and educational community and it could prompt new thinking and inspire others to build on it.

Attention to the origin, quality, and composition of all the materials and reagents that are commonly used in in vitro methods is essential to ensure data reproducibility and reliability, as advocated in GIVIMP.

Moreover, following the European Citizens' Initiative “Stop Vivisection” submitted to the European Commission (EC) in 2015 [22], the EC took several initiatives to accelerate the development and uptake of non‐animal approaches in research and testing. These initiatives included knowledge sharing, the creation of databases and inventories for finding information on knowledge sources related to the 3Rs, alternative methods and search principles [23], and the promotion of education and training activities [24]. Examples of available animal‐free alternatives to animal‐derived materials and reagents, along with some resources/references will be given later in the discussion section (Table 1).

**TABLE 1:** Main outcomes of the survey

Topic	Outcome
Most used animal‐derived reagents	Serum, antibodies and dissociation enzymes are the three most commonly used reagents based on animal ingredients
Main issues with the use of animal ingredients	Batch‐to‐batch variability or low reproducibility are the major issues, followed by ethical concerns
Consideration of non‐animal alternatives	Forty three percent of respondents considered the use alternatives to serum, mainly motivated by personal decision. Proportions of respondents who considered the use of any animal‐free alternatives were higher among biotechnology companies compared to other organizations.
Reasons for not considering the use of non‐animal alternatives	1 in 3 respondents were either not aware of the availability of animal‐free alternatives or preferred not to modify protocols already developed in their lab;
	Also high costs were considered an obstacle (by 21%).
Advantages of using non‐animal reagents	Most of respondents (76%) considered ethics as a major advantage of using alternative ingredients; more than half (51%) considered that increase of reproducibility as a major advantage
Perceived animal sufferance	Almost half of the survey participants answered that the production of serum or antibodies implies a very high or high level of sufferance for the animal (48% for serum and 44% for antibodies)
Lack of awareness about animal sufferance	Some respondents (8‐11%) believe that the level of animal sufferance is minimal for any of these reagents
Knowledge about availability of non‐animal ingredients	More than half of respondents rated their levels of awareness and knowledge on animal‐free alternatives as low or extremely low. The respondents from the industry, compared to other organization types, rated their awareness/knowledge higher, and researchers from translational/applied research rated their awareness/knowledge higher than these from basic/fundamental research. Students in particular were more likely to rate their awareness/knowledge as low or extremely low, compared to more senior colleagues.
Educational/professional experience on non‐animal ingredients	Most of respondents (68%) declared that their own level of information on animal‐free alternative materials and reagents was inadequate

In short, to replace proteins purified from animal tissues, such as bovine serum albumin, dissociation enzymes (usually porcine/bovine trypsin) or antibodies harvested from sera or ascites, it can be broadly recommended to use proteins produced recombinantly in vitro. In addition, generation of new antibodies is possible using fully animal‐free in vitro technologies such as phage display, which obviate the need for animal immunization [25]. Synthetic alternatives to Matrigel have been described [26]. As a starting point for the development of serum‐free media (either for each specific cell types or, ideally, for universal use), van der Valk 2022 [27] recommends a rich medium composed of equal volumes of DMEM and Ham's nutrient mixture F12, with a supplementation of recombinant insulin and transferrin plus the mineral selenium (ITS). For further improvement, growth factors, hormones, and proteins could be added [28]. A gradual/different replacement process may be required for different cell types [20]. Researchers may choose to use human‐derived products, such as human serum or human platelet lysate, the latter having been mostly used in the stem cells expansion [29]. However, to achieve more reproducible cell culture conditions, it is recommended to use a chemically‐defined medium, where the identity and concentration of all media supplements is known [28]. Alternatively to purchasing ready‐to‐use media, customized chemically‐defined medium can also be prepared in the laboratory itself [30] and thence adapted to the specific needs of the utilized cells. It should also be noted that “animal‐free alternatives,” such as chemically‐defined media or recombinant proteins, when consisting of defined reagents with a publicly disclosed and reproducible composition, can provide lower to no batch‐to‐batch variation than reagents purified from animals. Therefore, they do not only offer an alternative to animal use but also present scientific benefits, being in fact an improvement and therefore an advanced technique. This is particularly important considering today's research reproducibility crisis [31].

In an effort to get a better understanding of the state of use of animal‐derived materials and reagents and the level of knowledge on available non‐animal alternatives, Oltre la Sperimentazione Animale (OSA) [32] in collaboration with the Joint Research Centre (JRC) of the European Commission (EC) [33], Technical University of Denmark [34], Eurogroup for Animals [35], Deutscher Tierschutzbund e.V. [36], and the Centre for Predictive Human Model Systems [37], launched a global online survey aimed at understanding: 1) what are the most commonly used animal‐derived materials and reagents, 2) what are the main issues perceived as associated with the production and use of animal‐derived materials and reagents, 3) what is the current level of knowledge on available non‐animal alternative materials and reagents, and 4) what educational and information sources could be most useful or impactful to disseminate knowledge on non‐animal alternative materials and reagents. The survey was mainly addressed to scientists working on in vivo, in vitro, in silico methods, in academia as well as pharmaceutical or cosmetic companies. Results of the survey were used to identify the motivations and barriers related to the use of animal‐free reagents across these sectors, and to prioritize actions that can be taken to increase the awareness, knowledge, and use of such reagents. Here, the results of this survey are presented, along with a proposed list of prioritizable implementation strategies and possible target actions that we believe could help increase awareness and facilitate the acceptance and use of non‐animal reagents and materials whenever feasible. The ultimate goal is to encourage a shift toward a more reproducible, scientifically sound, and ethically sustainable research.

## 2 METHODS

The survey was conducted online between December 2020 and May 2021 using the European Union's survey management system, EU Survey. The URL to access the survey was disseminated through social media platforms, the creation of a promotional video, emails, international seminars, and collaborating partners’ websites.

The survey comprised 19 multiple‐choice questions divided into the following four main sections: a) personal information (4 questions); b) methodological approaches, materials and reagents (5 questions); c) awareness (6 questions); d) knowledge and information sources (4 questions) (see Supplementary file 1).

### 2.1 Audience selection criteria

In addition to the distribution via social media, YouTube, and other non‐targeted channels, we also employed a practical and simply replicable approach for using PubMed to generate large email lists of potential participants, as previously described [38]. The diverse combinations of search terms and Boolean operator used to generate the targeted email lists, are reported in the table in the Supplementary file 2.

### 2.2 Data analysis and presentation

Survey results were organized, analyzed, and plotted using Microsoft Office Excel, GraphPad Prism 8.4.3., and RStudio.

## 3 RESULTS

An average of 4500 emails for each combination of search terms have been sent. A total of 551 persons from 52 countries participated in the survey, which was launched on December 6^th^ 2020 and remained opened for 5 months. Below, the main outcomes of the survey are reported.

### 3.1 General information on survey participants

**FIGURE 1:**
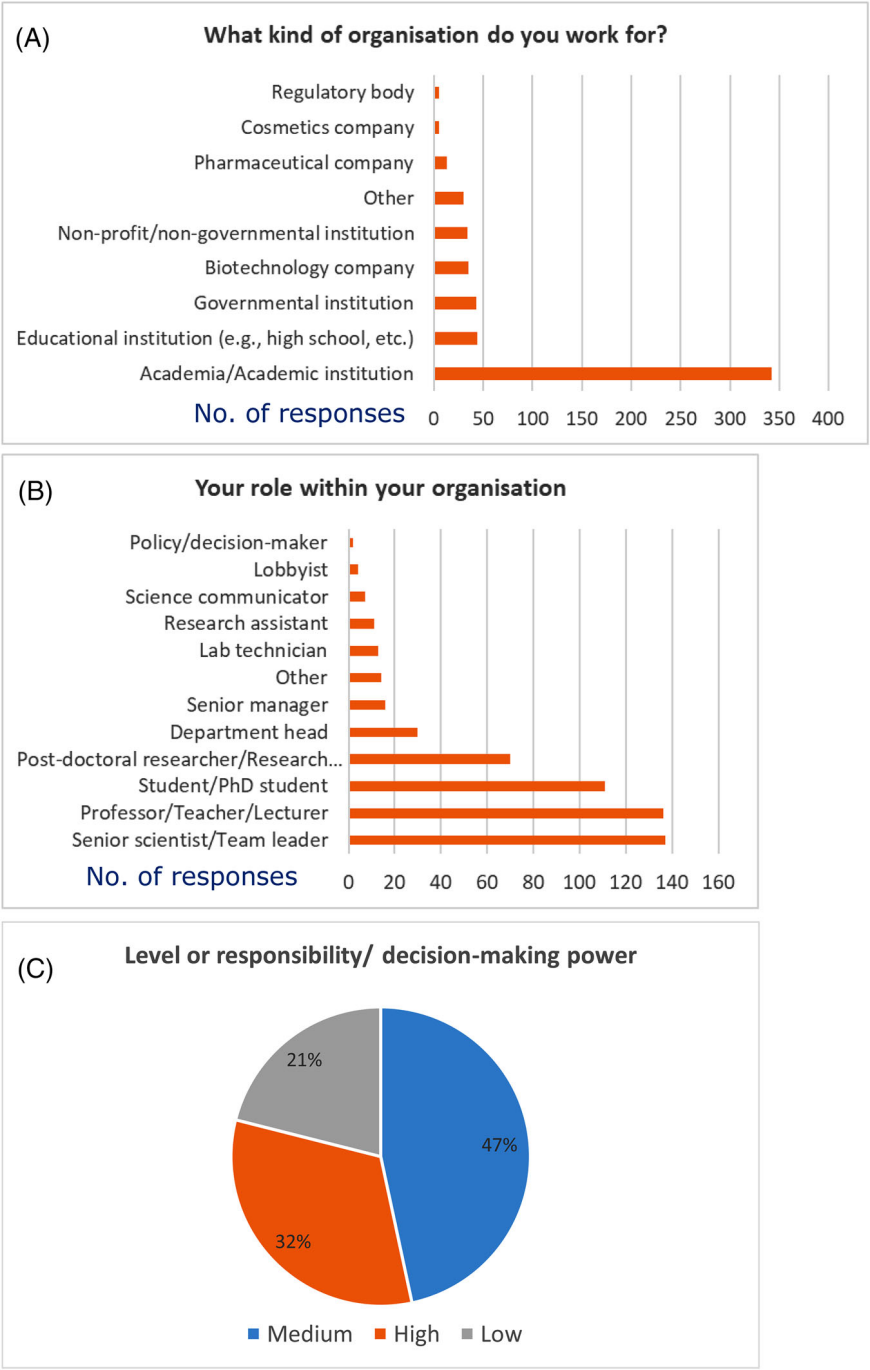
Respondents’ organizations (A), their role (B) and their level of responsibility within their organization (C). Graphs in A) and B) show absolute number of replies. C) shows percentages of replies

Questions 1 to 4 aimed to gather general information about survey participants. With regards to their affiliations, the highest majority (62%, 342 in 551) work in academia, an equal 8% work either in educational (e.g., high schools) or governmental institutions, and an equal 6% in either biotechnology companies or in non‐profit/non‐governmental institutions (Figure 1A).

An equal 25% of participants are senior scientists/team leaders (137 in 551 respondents) or professors, teachers, or lecturers (136 in 551). Twenty percent (111 in 551) are students or PhD students, while 13% are post‐doctoral researchers or research fellows (70 in 551 respondents) (Figure 1B).

Participants were also asked to rate their level of responsibility within their organization. Forty‐seven percent (257 in 551) rated their responsibility in decision‐making power as “medium,” 32% (178 in 551) as “high” and 21% (116 in 551) as “low” (Figure 1C).

**FIGURE 2:**
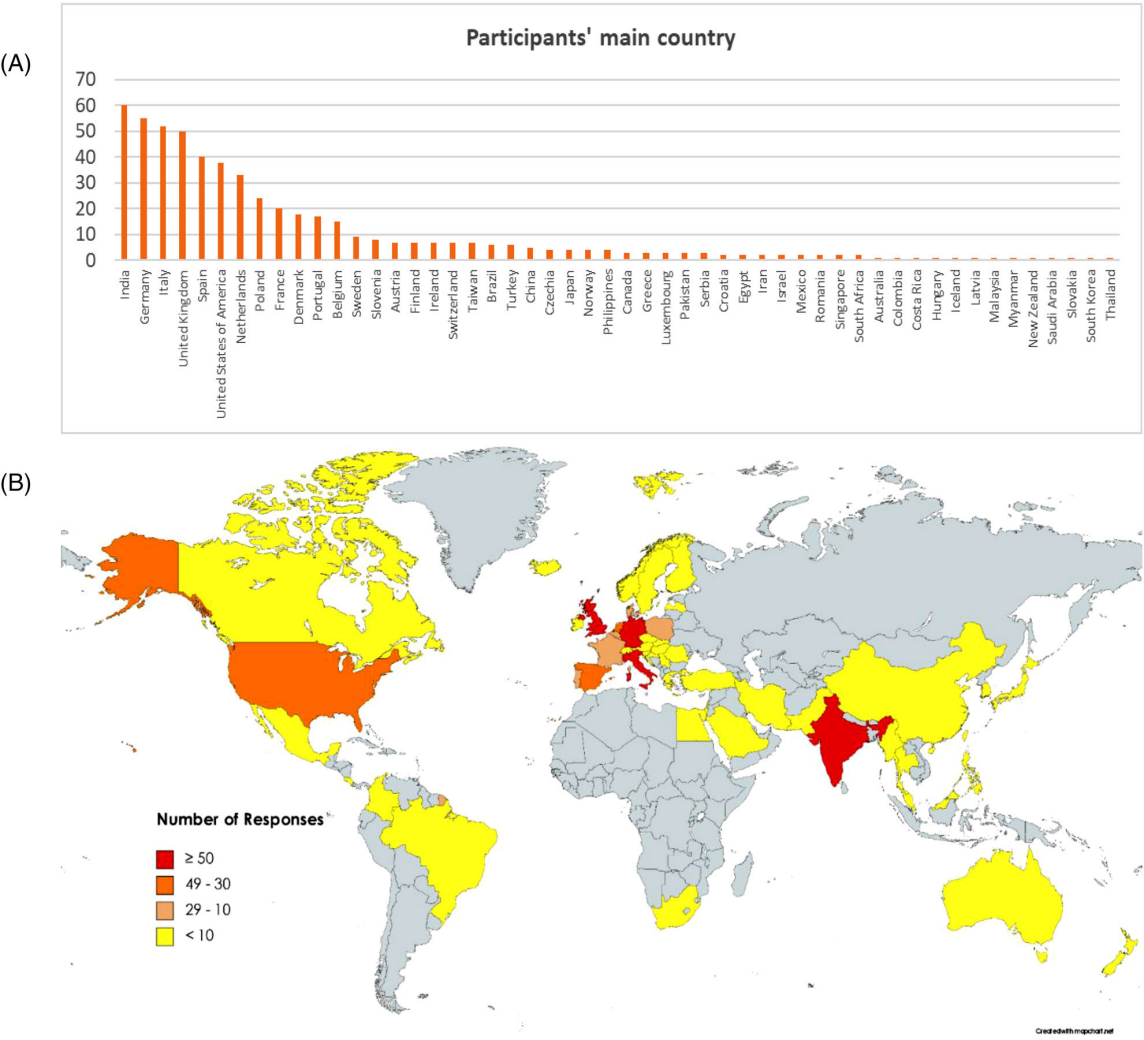
Main country of origin of survey respondents (A and B). Graph in a shows absolute number of replies. https://mapchart.net/terms.html

Eleven percent of participants are from India (60 in 551), followed by Germany (10%), Italy and the United Kingdom (both 9%), Spain and the United States (both 7%), and the Netherlands (6%). Other countries are indicated in Figure 2.

### 3.2 Methodological approaches, materials and reagents

**FIGURE 3:**
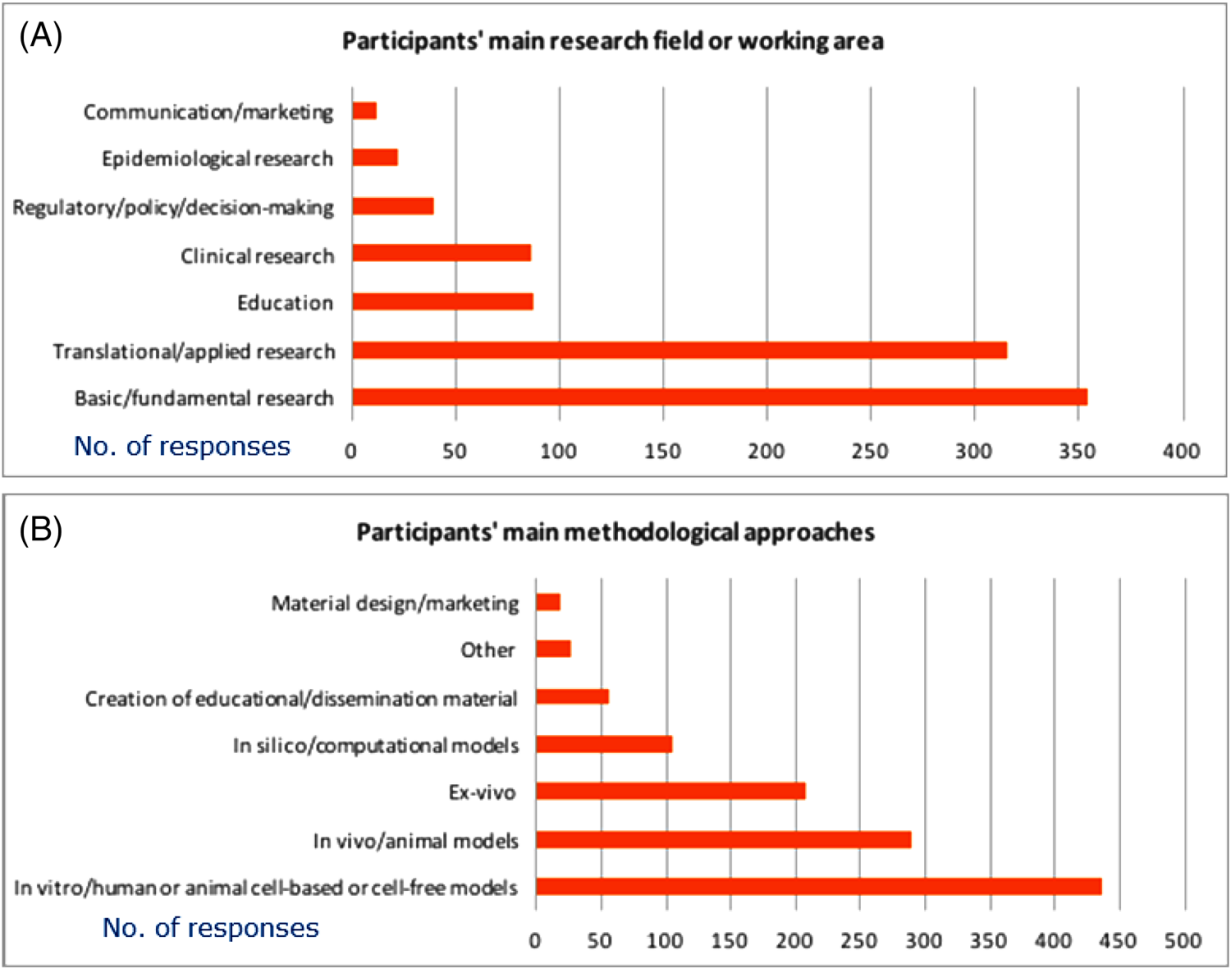
Respondents’ main fields of research or professional activity (A) and main methodological approaches (B). Respondents could select more than one option in both cases. Graphs show absolute number of replies

Sixty‐four percent of participants indicated basic/fundamental research as their main research field, 57% indicated translational/applied research, an equal 16% indicated to work on education or clinical research, while 7% work on regulatory, policy and decision‐making (Figure 3A, participants could select more than one option).

A large proportion of respondents (79%) indicated that they mainly work with in vitro (human and/or animal) cell‐based or cell‐free models; 53% with in vivo and animal models; 38% with ex vivo models; 19% with computational models, while 10% work on the creation of educational or dissemination material (Figure 3B). Also in this case, participants could select more than one option.

**FIGURE 4:**
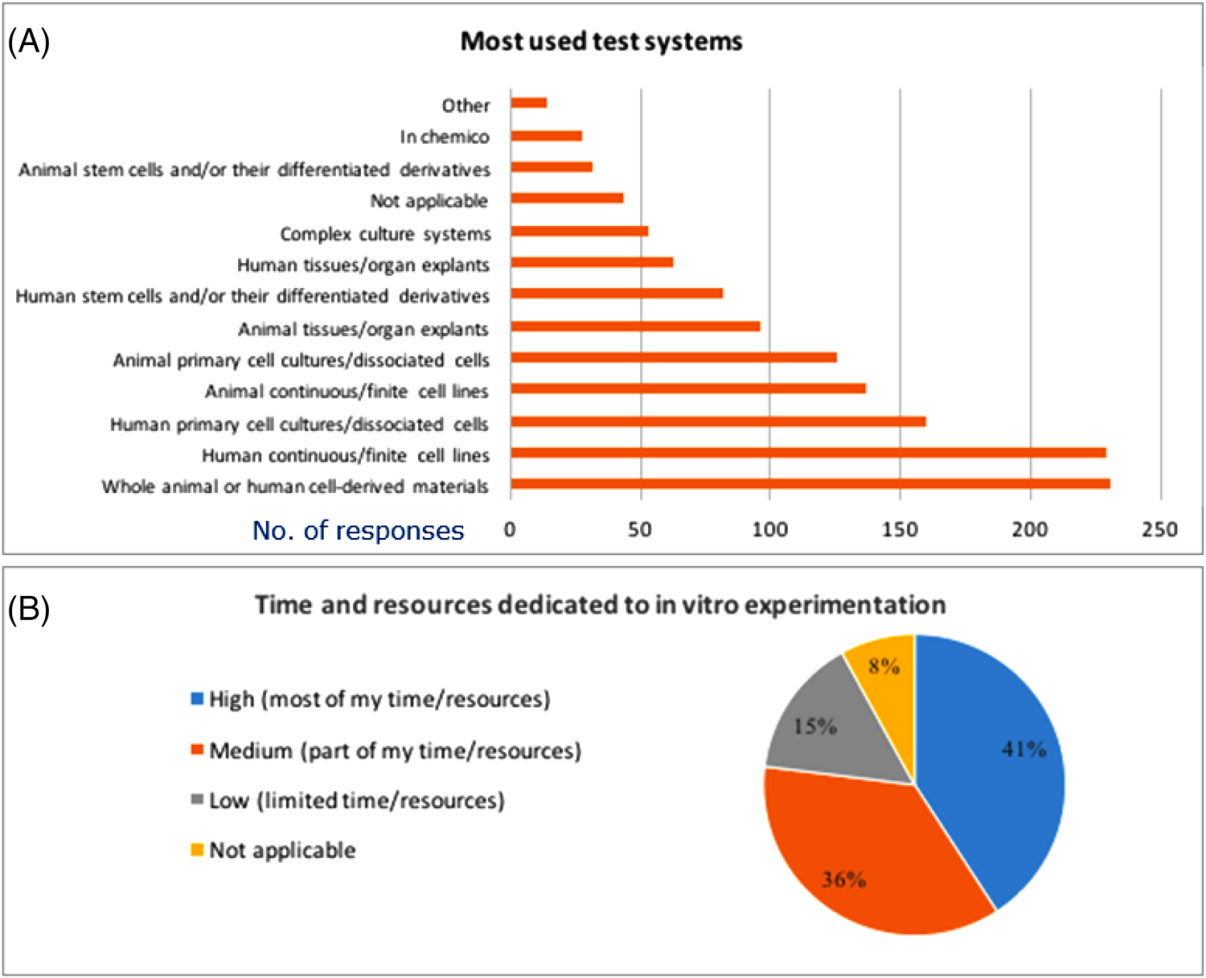
Most used test systems (A) and time and resources dedicated to in vitro experimentation (B). In A) respondents could select more than one option, and graph shows absolute number of replies. B) shows percentages of replies

Among survey respondents, the most used test systems are human continuous or finite cell line and whole animal‐ or human cell‐derived materials (42% of replies for both groups). Regarding the latter option, it should have been considered at the stage of designing survey questionnaire to separate animal and human‐cell derived materials. Since these two options were kept together, it is not possible to retrieve the exact number of respondents who used whole animals to derive cell materials. Moreover, 29% of respondents used human primary cell cultures or dissociated cells, 25% animal continuous or finite cell lines, 23% used animal primary cell cultures and dissociated cells and other test systems, as indicated in Figure 4A (also in this case, respondents could select more than one option). Moreover, 41% of participants (225 in 551) declared that they dedicate a high proportion of their time and resources on in vitro experimentation, 36% (199 in 551) only part of their time and resources, while a more modest 15% (83 in 551) allocate only a limited part of their work on in vitro experimentation (Figure 4B).

**FIGURE 5:**
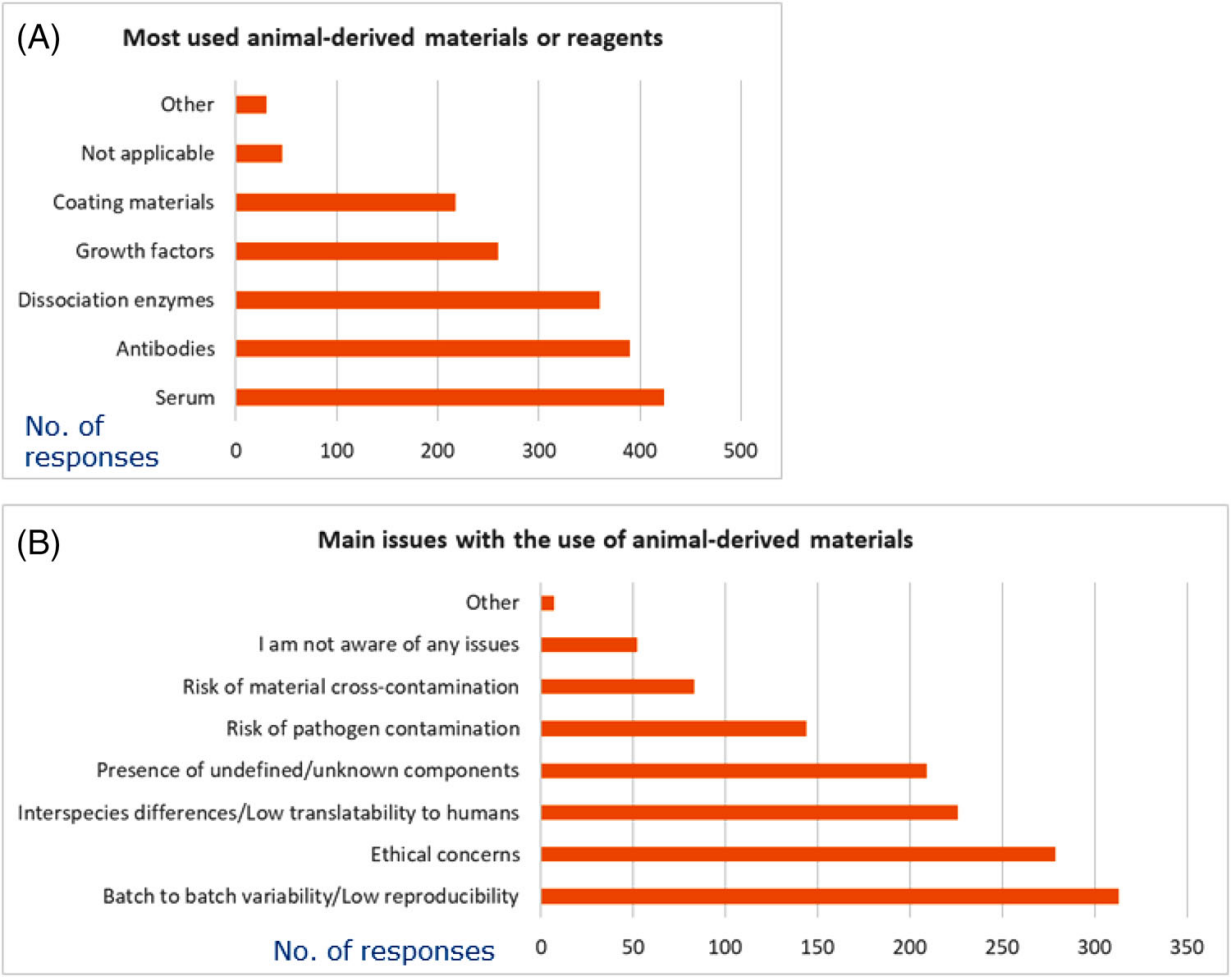
Most used animal‐derived materials or reagents (A) and main issues associated with their production and use (B). Respondents could select more than one option; graphs show absolute number of replies

Animal serum was indicated by the vast majority of participants (77%) as the most used animal‐derived reagent, followed by animal‐derived antibodies (71%), dissociation enzymes (e.g., trypsin, collagenase, papain, nucleases, etc.) (65%), cellular growth factors (47%) and coating materials, such as matrigel or laminin (40%) (Figure 5A, respondents could select more than one option).

### 3.3 Awareness

The survey also aimed at identifying the main issues perceived by the respondents toward the use of animal‐derived materials and reagents. The majority of survey participants (57%) responded that batch‐to‐batch variability or low reproducibility was the major deterrent, followed by ethical concerns (51%), interspecies differences (41%), presence of undefined components (38%), risk of pathogen contamination (26%), and risk of material cross‐contamination (15%). Notably, 9% of respondents were not aware of any issues (Figure 5B).

**FIGURE 6:**
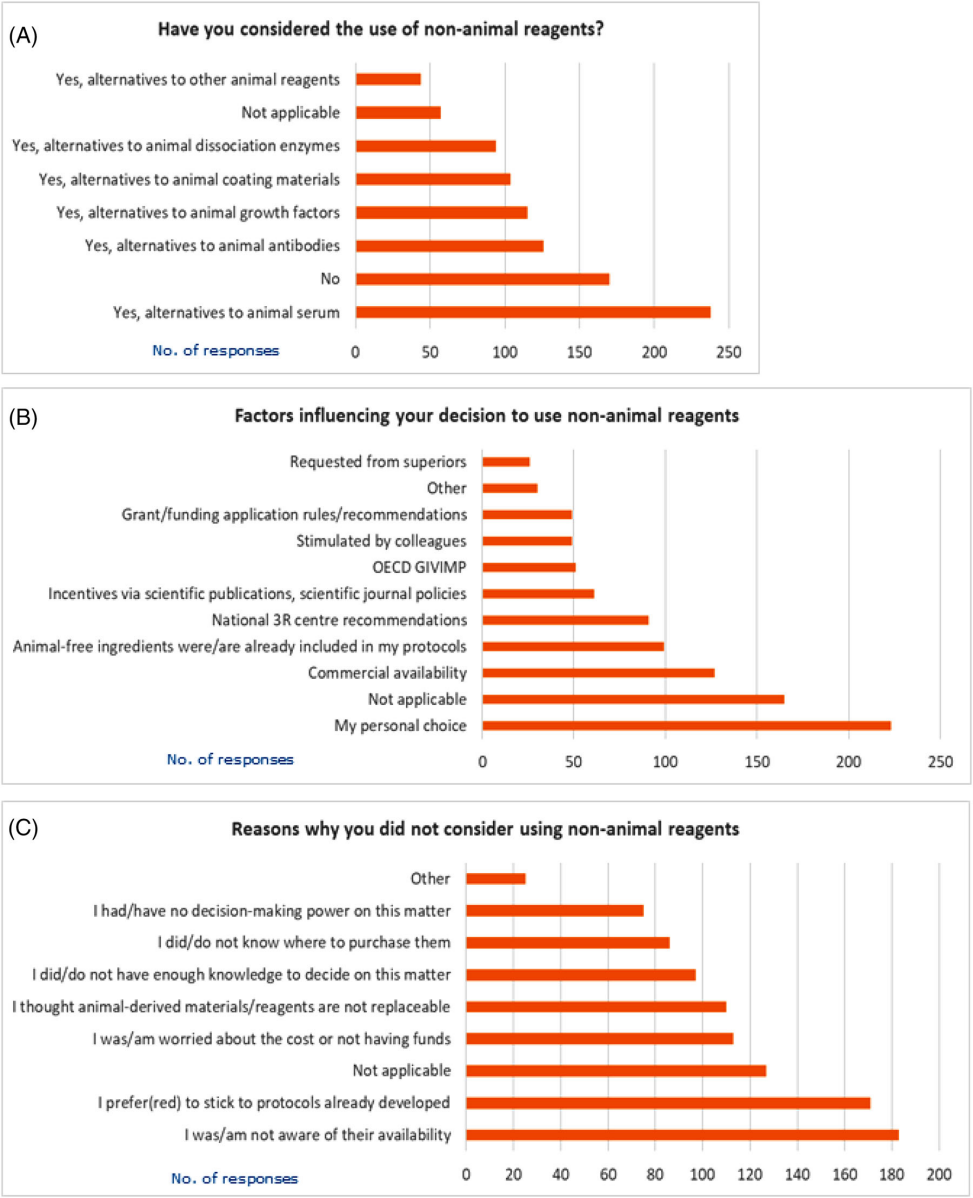
Considerations about the possibility to replace animal‐derived ingredients with non‐animal alternatives (A and B) and reasons for not considering these alternatives (C). Respondents could select more than one option; graphs show absolute number of replies

Participants were also asked if they ever considered using any animal‐free alternative. Forty‐three percent of respondents considered the use of non‐animal alternatives to animal serum, followed by non‐animal antibodies (23%), growth factors (21%), coating materials (19%), and dissociation enzymes (17%). Around 31% of the participants responded that they never considered the use of non‐animal derived materials (Figure 6A). Disaggregated analysis (see Supplementary file 3) revealed that among all types of organizations, biotechnology companies had the highest proportion of respondents (77%), who considered the use of at least one animal‐free alternative to the animal‐derived ingredients, which they normally work on (Fig. S1). As regard to the relationship between the role within one's organization, smaller percentage of students (about 46%) considered the use of any animal‐free alternatives, compared to post‐doctoral researchers (56%), senior scientists or team leaders (66%), and professors/teachers/lecturers (70%) (Fig. S4).

It was also shown that 83% of respondents working with stem cells considered the use of animal‐free alternatives, compared to 60% of all survey respondents (Fig. S10). There were no notable differences in this answer among participants working solely in basic/fundamental research as opposed to translational/applied research (Fig. S7).

An attempt was also made to understand the main reasons underlying the decision to consider non‐animal alternatives. Forty percent of respondents decided to choose non‐animal alternatives for personal reasons. Commercial availability of non‐animal reagents (23%), existing use of non‐animal alternative‐related protocols in the lab or institute (18%), recommendations from national 3R centres (17%), and policies or incentives by scientific journals (11%) represented other popular reasons to opt for non‐animal alternatives. An equal 9% based this decision considering GIVIMP indications, inputs from colleagues, or to abide to recommendations in grant funding applications. Finally, 5% of respondents declared they were requested by superiors to use non‐animal alternatives (Figure 6B).

On the other hand, lack of awareness regarding their availability was the most prominent reason for not considering animal‐free alternatives (33%), followed by preference towards existing protocols developed in the lab (31%), high costs or lack of funding (21%), the belief that animal‐derived materials were irreplaceable (20%), lack of sufficient information to make an informed choice in this matter (18%), lack of knowledge on where to procure non‐animal based materials (16%), and lack of decision‐making power by participant (14%) (Figure 6C).

**FIGURE 7:**
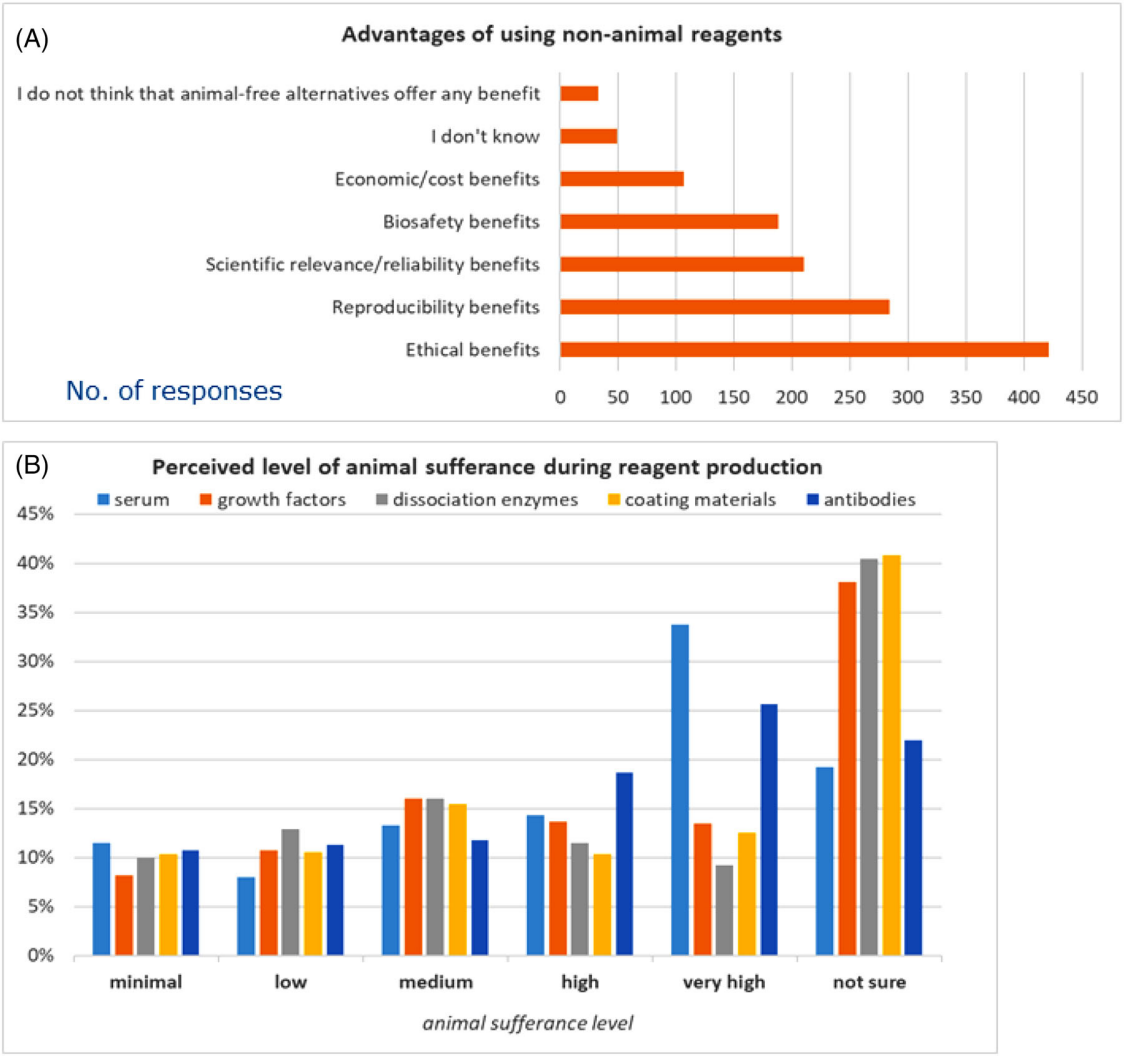
Advantages of using non‐animal alternatives (A) and perceived level of animal sufferance (B). In A) respondents could select more than one option and graph shows absolute number of replies. B) shows percentages of replies

Participants were asked to identify one or more advantages of using non‐animal reagents instead of animal‐derived ones. Most of respondents believe that the benefits would be mainly of ethical nature (76%), or linked to increase of reproducibility (51%). Furthermore, other advantages are the higher scientific relevance or reliability of non‐animal alternatives (38%), improved biosafety (34%) and lower costs (19%). Nine percent declared they did not know what the benefits could be, while 6% of participants believe that animal‐free alternatives offer no benefits (Figure 7A).

Moreover, participants were asked to attribute a score ranging from 1 to 5 to the level of suffering believed to be perceived by animals during the preparation of some of the most common animal‐derived reagents, where 1 means “minimal level of sufferance” and 5 “high level of sufferance.” It emerged that for a half of the survey participants, the production of serum and antibodies implies a very high or high level of sufferance for the animal (53% and 49%, respectively). About reagents such as growth factors (38%), dissociation enzymes (40%), and coating materials (41%) there is a greater uncertainty about the level of distress perceived by the animal. Notably, between 8% and 11% of respondents believe that the level of animal sufferance is minimal for any of these reagents (Figure 7B).

### 3.4 Knowledge and information sources

The last group of questions aimed to investigate the perceived level of knowledge and the adequacy of information/education received on currently available animal‐free alternatives materials and reagents for in vitro experimentation, as well as to understand what type of educational and dissemination sources could be more useful for spreading knowledge.

**FIGURE 8:**
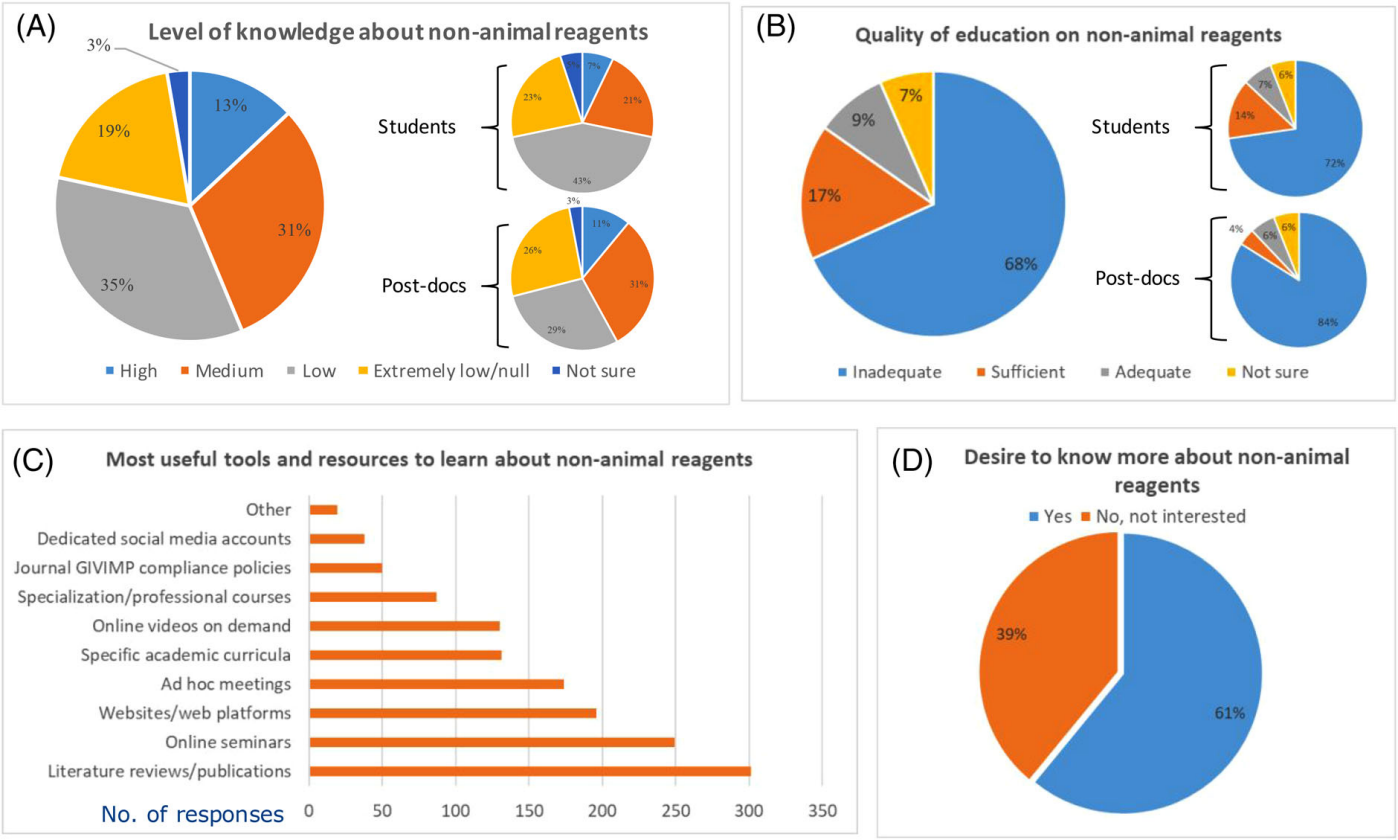
Level of knowledge (A), quality of education (B), tools for knowledge sharing (C) and interest to know more about non‐animal alternatives (D). In A and B, disaggregated data are also shown for the groups of students and post‐docs. In C) respondents could select more than one option and graph shows absolute number of replies. In A), B), and D) percentages of replies are shown

Thirty‐five percent of participants rated their level of awareness or knowledge about currently available animal‐free alternatives as “low,” 31% as medium, 19% as “extremely low/null,” while a lower group, 13% of respondents, think their level of knowledge as “high.” The remaining 3% were “not sure” (Figure 8A). Notably, students (111 respondents) and post‐docs (70 respondents) more frequently rated their level of awareness on alternatives as extremely low or low compared to senior scientists, teachers, professors, or lecturers (Figure 8A).

Moreover, higher proportion of respondents from the biotechnology companies or industry (pooled answers from biotechnology, pharmaceuticals, and cosmetics) rated their awareness of knowledge on the currently available animal‐free materials as high (20% only biotechnology companies, 15% all industries) or medium (37% only biotechnology companies, 40% all industries) compared to respondents from other institutions. For example, only 10% of respondents from academia rated their awareness/knowledge as high and 30% as medium (Fig. S2). Regarding particular research fields, respondents dedicated to translational/applied research, compared to these dedicated to basic/fundamental research, more frequently rated their level of awareness or knowledge on animal‐free alternatives as high (15% translational researchers vs 10% basic researchers) or medium (34% of translational researchers vs. 27% of basic researchers) (Fig. S8). Twenty two percent of surveyed researchers using stem cells as one of the main test systems declared high awareness or knowledge on the animal‐free alternatives, while on average 13% of all survey respondents gave this answer (Fig. S11). It was also notable that compared to the average respondent, a subgroup that was not aware of the issues associated with the animal‐derived ingredients (Figure 5B) more frequently also indicated low or extremely low levels of awareness on the animal‐free alternatives (Fig. S13). Concerning education received, 68% of respondents judged their own level of information on animal‐free alternative materials and reagents received during their own academic and/or professional experience as “inadequate”; 17% considered it “sufficient,” 9% judged their level of information as “adequate,” while 7% of participants were “not sure” about the answer (Figure 8B). Also in this case, a higher proportion of students and post docs rated their level of education on alternatives as inadequate, compared to senior scientists, teachers, professors, or lecturers (Figure 8B, Fig. S5). Among the types of organizations, respondents working in governmental institutions less frequently rated the education received as inadequate (51%), compared to 68% of all respondents (Fig. S3). There were no major differences in the answers to this question between basic/fundamental research scientists and applied/translational research scientists (Fig. S9). Slightly higher proportion of stem cell scientists (Fig. S12) rated the level of information received as adequate (13%) or sufficient (19%), compared to all pooled respondents (9% adequate, 17% sufficient). Compared to the average respondents, those who never considered the use of alternatives or that were unaware of the issues associated with animal‐derived materials and reagents, more frequently also indicated an inadequate level of knowledge received (Fig. S14).

Regarding the tools and resources to learn about non‐animal alternatives, 55% of participants indicated that “literature reviews or publications” are the most useful or impactful educational and dissemination sources, 45% suggested “online seminars,” 36% “websites/web platforms,” 32% “ad hoc meetings” (e.g., conferences, summer schools, workshops), an equal 24% believed that specific academic curricula and “online videos on demand” would be the best options, 16% opted for “specialization or professional courses,” 9% “Journal GIVIMP compliance policies,” and 7% suggested that “dedicated social media accounts” represent the most useful or impactful educational and dissemination sources to share knowledge about animal‐free alternatives (Figure 8C).

Finally, 61% of respondents expressed their interest to know more about animal‐free alternatives, while 39% declared they were not interested (Figure 8D). Notably, the percentage of interested people was higher (69%) for both the groups of students and post‐docs (Fig. S6). Respondents, who previously declared no awareness of the issues associated with the animal‐derived ingredients, were substantially less likely to answer that they would like to know more about the animal‐free alternatives (Fig. S15).

The main outcomes of this survey are summarized in Table 1.

## 4 DISCUSSION

This survey aimed to map the current state of use of animal‐derived reagents across different sectors and to understand what obstacles may hamper the implementation of non‐animal derived alternatives.

Of the 551 survey participants, most work in academia, while the number of responses from industry/private sector is relatively low (about 6%). There were only 35 replies from the biotechnology companies, 13 from pharmaceutical companies, and 5 from cosmetics companies. Perhaps, this could be due to the fact that our survey dissemination strategy was more efficient at reaching respondents working in academic institutions, if academic researchers were more likely to be corresponding authors on published scientific articles that we targeted. In potential follow‐up activities, it should be considered how to include these groups more efficiently by the dissemination strategy. Most of the total responses were obtained from India, Germany, Italy, and the UK. Unfortunately, not many responses came from countries like China, Australia, or Russia, where we would expect to have a similarly strong participation. Also, a relatively small sample comes from the USA, where the number of responses is almost equal to Spain or Netherlands. In addition, France was also not strongly represented in the survey.

Despite these (external) reporting drawbacks, the results of this survey (summarized in Table 1) point to some important directions. The significant lack of knowledge about the possibility to replace animal‐derived ingredients with non‐animal alternatives (about a third of survey participants), points to the importance of investing in activities aimed at raising awareness about the existence of animal‐free alternatives. In particular, students and post‐docs more frequently stated their knowledge and education on non‐animal ingredients as low or inadequate (Fig. S5), and expressed a high level of interest to know more about the topic (Fig. S6). Therefore, possible dissemination initiatives could be designed to specifically target these groups. Moreover, the fact that several responders did not know if a suitable alternative was available for their purpose, brings about the recommendation to increase the accessibility of finding animal‐free alternatives, for example through dedicated websites (e.g., https://fcs‐free.org/). Another obstacle identified is the cost of replacing animal‐derived ingredients (21% of participants), which could be offset by providing dedicated funding to projects with this particular aim. Examples of existing activities in line with these recommendations are described in the following sections.

### 4.1 Increasing awareness about non‐animal products/ingredients

Awareness is fundamental in bringing about any change. Despite the fact that scientists strive for progress and innovation, it is a well‐known phenomenon that they also operate in research silos [39], and are predominantly exposed to new information emerging in their respective research fields. The use of serum‐free cell culture conditions is perhaps nothing new for stem cell scientists, biotechnologists or researchers working with biopharmaceuticals, where such approaches have been developed for many years now and/or are obligatory. The awareness of the risk of unintentional introduction of contaminants via raw materials from animal sources has led to a move toward animal‐free medium components and adaptation of cell lines grown in animal‐free media for the production of biologics and vaccines. Indeed, compared to other types of organizations surveyed, respondents from biotechnology companies rated higher their awareness and knowledge on animal‐free alternatives and a larger proportion of them considered the use of animal‐free alternatives (Fig. S2). Translational/applied researchers were also more likely to rate their awareness and knowledge on alternatives as high, compared to basic/fundamental research scientists. Compared to the pooled survey participants, a subgroup of researchers working with stem cells as a test system had larger proportions of respondents who already considered the use of animal‐free alternatives or rated their awareness on these alternatives as high. This example may be reflecting how the benefits associated with the use of animal‐free alternatives fuelled the use of these materials in certain research areas. However, in other disciplines researchers may not be aware of the existence of chemically‐defined serum‐free media. It is therefore important to initiate activities that can span multiple research fields (including applied/translational research or stem cell research, where despite positive trends described, still a half of respondents rated their awareness as low or extremely low), and to seed the awareness across multiple groups. One of such activities was this survey itself. Through answering the questions on the use of animal‐derived ingredients, participants were encouraged to reflect on their research activities. The visibility of alternatives to animal‐derived ingredients can be increased by presentations, online seminars [11] and publications describing development of protocols for animal‐free alternatives. In 2020, freely accessible webinar series on non‐animal derived affinity reagents was co‐organised by the US National Toxicology Program Interagency Center for the Evaluation of Alternative Toxicological Methods (NICEATM), the PETA Science Consortium International e.V., and the EURL ECVAM [40]. Along the same line, a very recent report published by EURL ECVAM presents a systematic mapping of the animal‐free alternatives currently available to replace the animal‐derived ingredients included in a battery of in vitro methods suitable to assess thyroid signaling disruption (here presented as a case study), and provides some insights about their accessibility and possible replacement strategies [41].

Changes do not only come from the outside, there also has to be pressure from within: students' expectations continue to be a driver in the evolution of animal‐welfare education [42]. Our survey analysis also showed that out of 111 students/PhD students who participated in the survey, about 69% expressed an interest to know more about non‐animal ingredients and reagents.

The ethical question, if and how animals (and animal‐derived products) should be used, has to be asked in the university laboratories as well. However, when awareness of animal‐free reagents is already low in training facilities and therefore animal‐derived reagents are being portrayed as unavoidable, then scientific curiosity and creativity would be diminished. Thus, education and training sessions at universities will break up the lack of awareness in future generation of scientists and open the gate to new inventions and discoveries.

Another strategy leading to increased awareness is by instructing researchers to report the use of animal‐derived ingredients in in vitro methods and to provide a comment if animal‐free adaptation was pursued. Such reporting requirement stimulates the reflection on research practices. Author submission guidelines in the journal ALTEX state that “*ALTEX recommends the substitution of all materials that are obtained or derived from animals subjected to pain or suffering. (…) we require authors to discuss this issue, preferably in the Discussion section, and indicate whether such materials could/shall be replaced in future studies*.” [43] Similarly, according to the GIVIMP document, the use of animal sera in cell culture medium is discouraged and GIVIMP reporting requirements include the disclosure about using animal sera. In some funding applications, the use of animal‐free ingredients is one of the criteria for awarding the funding, for example, the terms and conditions of Animal Free Research UK funding calls do not allow the use of serum or polyclonal antibodies. Applicants for the € 20.000 Herbert Stiller Research Grant for the development of animal‐free assays, must ensure in their grant application that none of the materials used are derived from animals [44]. Proefdiervrij, a Dutch society for the replacement of animal testing, supports projects that meet particular criteria including the avoidance of FBS whenever it is possible [45]. The ECEAE (European Coalition to End Animal Experiments) is calling for applications for a prize for animal‐free antibodies. It demands that the project must not involve any animal experiments or animal‐derived products [46]. Setting such requirements provides an incentive to reflect on the use of animal‐free reagents at the early stages of project planning and increases the awareness about their importance among in vitro scientists seeking to obtain funding.

Furthermore, an immense step forward could be achieved if peer‐reviewers would demand to verify experimental results in a xeno‐free or chemically defined environment [47]. Such a step is in the very own interest of reviewers, applicants and journals as it can ensure reproducibility for published results.

### 4.2 Improving accessibility of reagents and protocols

The results of this survey clearly identified that some researchers are not confident whether the alternatives for the animal‐derived ingredients exist, and where they can be found (Table 1). When such chemically‐defined media, or other animal‐free alternatives exist on the market, they are not always supported by independent validation published by the end users. Furthermore, commercial suppliers do not always disclose the need for all necessary supplements or the most suitable adaptation protocols. Depending on the individual attitudes of their representatives, additional support can be obtained upon request. Altogether, collecting information about accessibility of certain reagents and necessary details for their correct use requires investment of time, and often expertise. One of the tools created to facilitate the search for the replacement of serum in tissue culture is the FCS‐free database [48]. It would be helpful if similarly well‐maintained and curated databases existed for other animal‐derived ingredients, such as proteins, including dissociation enzymes and antibodies. The UK National Centre for the Replacement Refinement and Reduction of Animals in research (NC3Rs) [49] provides a list of animal‐free reagents and resources [50]. PETA has prepared a useful website listing the suppliers of animal‐free antibodies [40]. The Xeno Free Initiative is working on an online toolkit to create a central hub and open resource repository for scientists to develop as well as adopt research approaches that are free of animal‐derived reagents and media [51]. To facilitate access to information on the animal‐free in vitro reagents and protocols, it is also recommended that the end users can easily share their experience in this field. Currently, one example of such approach is Serum‐free cell and tissue culture LinkedIN group [52]. One could envisage similar type of fora providing space for users to share their experience and protocols about other animal‐free protocols. This could allow for the knowledge transfer to proceed in a quicker and more direct manner – particularly for negative results, which are less likely to be published – and thereby avoiding delays in dissemination of optimisation experiments on animal‐free alternatives. An exceptional example is the peer‐reviewed open access journal Antibody Reports, created in association with Geneva Antibody Facility [53], which creates a platform to publish the results (also, negative ones) of the validation studies using recombinant antibodies. The journal, is part of a broader project, which aims to promote the widespread use of recombinant antibodies by academic researchers and, ultimately, to promote the replacement of animal‐derived antibodies. The project also comprises the AntiBodies Chemically Defined Database (ABCD) [54], a manually curated archive of sequenced antibodies, developed by the Geneva Antibody Facility at the University of Geneva (Switzerland). Several examples of available alternatives for the most commonly used animal‐derived ingredients, along with associated resources are summarized in Table 2.

**TABLE 2:** Examples of available animal‐free alternatives to animal‐derived materials and reagents, along with some resources/references

Animal‐derived materials/reagents	Animal‐free alternatives	Possible resources/references
Animal sera	Serum‐free culture media and media supplements	FCS‐free database [48]
		NC3Rs ‐ Animal‐free in vitro technologies [50]
		Optimization of chemically defined cell culture media–replacing fetal bovine serum in mammalian in vitro methods [28]
		Alternative to FBS in animal cell culture ‐ An overview and future perspective [63]
		PETA list of Non‐Animal Cell Culture Products and Applications [64]
Animal‐derived antibodies	Recombinant animal‐free antibodies	Geneva Antibody Facility [53]
		AntiBodies Chemically Defined Database (ABCD) [54]
		PETA list of animal‐free antibodies suppliers [40]
		NC3Rs ‐ Animal‐free in vitro technologies [50]
Dissociation enzymes (e.g., porcine/bovine trypsin)	Recombinant dissociation enzymes (e.g. TrypZean®, TrypLE™) or non‐enzymatic agents	TrypZean™: An Animal‐Free Alternative to Bovine Trypsin [65]
		Tissue dissociation and primary cells isolation using recombinant collagenases class I and II [66]
		SciPro Recombinant AOF Tissue Dissociation Enzymes [67]
		BI Recombinant Trypsin Solutions [68]
		Amsbio Animal‐Free GMP Grade Collagenases and Neutral Protease [69]
		Adaptation of the HEp‐2 cell line to totally animal‐free culture systems and real‐time analysis of cell growth [70]
		NC3Rs ‐ Animal‐free in vitro technologies [50]
Coating materials /extracellular matrix components /basement membrane preparations (e.g., Matrigel™, collagen, gelatin, laminin)	Synthetic materials and recombinant proteins	Synthetic alternatives to Matrigel [26]
		Clean bioprinting ‐ Fabrication of 3D organ models devoid of animal components [71]
		Recombinant collagen for animal product‐free dextran microcarriers [72]
		NC3Rs ‐ Animal‐free in vitro technologies [50]
Animal‐derived growth factors, proteins	Human recombinant growth factors, proteins	How to choose your recombinant proteins, cytokines & growth‐factors [73]
		Recombinant Proteins (R&D Systems) [74]
		Recombinant Proteins (BIC) [75]
		NC3Rs ‐ Animal‐free in vitro technologies [50]

### 4.3 Increasing funding for the replacement of animal‐derived products and ingredients

Some researchers may be discouraged to replace animal‐derived ingredients in their labs due to perceived increased cost associated with this switch (21% of survey respondents expressed this concern) (Table 1). Indeed, some of the animal‐free alternatives, especially recombinant proteins, can be several‐fold more expensive than proteins purified from tissues (e.g., albumin or trypsin). However, the same is not necessarily true for other ingredients. Recombinant antibodies are in a similar price range as monoclonal antibodies made by hybridoma technology and by being completely sequence defined, they offer significant advantage over monoclonal and polyclonal antibodies. The price of chemically defined media is usually higher than of standard basal media but the numbers become similar when one considers the additional cost of FBS supplementation, comprising the majority of the total price. Chemically defined media can be associated with higher costs if coating or additional supplements are needed. Importantly however, even if the cost of a defined non‐animal derived ingredient is higher per purchase, one should consider that increased batch‐to‐batch reproducibility may in the long term reduce the waste of resources associated with irreproducible experiments [31].

Nevertheless, if any methods have been developed and optimized based on the historical data or protocols using animal‐derived ingredients, additional investment will be required to validate the method involving animal‐free alternatives. This aspect could be one of the causes for the participants of this survey to state that they prefer to stick to the protocols already developed in their labs (31% of participants). This is particularly relevant for formulating customized chemically defined media, which can become a substantial method development project in itself. Therefore, to increase the uptake of fully animal‐free approaches in in vitro methods, it is recommended to allow for dedicated funding sources for such endeavors. Examples include the UK NC3Rs CRACK IT Challenge “Animal‐free in vitro.” [55] This initiative, sponsored by Unilever and AstraZeneca, aims to support fully animal‐free adaptation of two in vitro methods, already validated as OECD test guidelines. The goal is to replace animal‐derived cell lines and develop protocols free of animal‐derived materials (especially, serum, antibodies, and metabolic enzymes), and to include these adapted protocols as an Annex to the current test guidelines.

### 4.4 Outlook

Considering the main results of the survey and the aforementioned initiatives that already are (or could be) put in place to increase awareness and incentivize the acceptance and use of non‐animal alternative products/ingredients, a list of proposals was identified, as summarized in Table 3. Several of these initiatives will be possibly implemented by OSA [32] and the other partners involved in this activity in collaboration with other relevant stakeholders.

**TABLE 3:** Proposals to increase awareness and promote the use of non‐animal products and ingredients, along with the description of resources required/Likely impact, and possible organization(s) which could take‐ or is/are already taking the action. Actions are ordered by priority

Priority	Action	Resources required/Likely impact	Possible organization(s) which could take‐ or is/are already taking‐ the action
1	Promote activities aimed at raising awareness about the existence of animal‐free alternatives (e.g., presentations, online seminars, publications, webinars), with a focus on students and post‐docs	Adequate expertise / Increased awareness; to break up the lack of awareness in future generation of scientists and open the gate to new inventions and discoveries; Creating expertise	Academia; EURL‐ECVAM [23, 24]; OSA [32]; CPHMS [37]; Deutscher Tierschutzbund e.V. [36]; NC3Rs [49]; PETA [40]
2	Increase accessibility of finding animal‐free alternatives through well‐maintained and curated databases	Adequate funding; Investment of time / Increased use of animal‐free alternatives	Geneva Antibody Facility [53]; NC3Rs [49]; EURL‐ECVAM [23, 24]
3	Improve education on non‐animal alternative products/ingredients by means of dedicated education and training sessions and the design of curricula at university	Expertise / Increased awareness on the limitations of animal‐derived components and the advantages of animal‐free, chemically defined materials and reagents; Creating new expertise	Academia; NC3Rs [49]; EURL‐ECVAM [23, 24]; CPHMS [37]
4	Include mandates in publications and research proposals to report the use of animal‐derived products/ingredients, and to comment when animal‐free adaptation was pursued	Expertise / raised awareness	Academia; Funding bodies; NC3Rs [49]
5	Incentivize activities aimed at validating non‐animal products/ingredients and protocols based on their use	Funding; Expertise; Awareness / Increased use of animal‐free products and ingredients	EURL‐ECVAM [23, 24]; NC3Rs [49]
6	Create dedicated social network platforms to enable end‐users to exchange experiences and protocols on the use of animal‐free alternatives	Awareness; Consistent use of animal‐free materials and reagents / Knowledge transfer; Avoiding delays in dissemination of optimisation experiments on animal‐free alternatives; Further increasing of the use of animal‐free and chemically defined products	Geneva Antibody Facility [53]; Academia
7	Create dedicated peer‐reviewed journals to publish results (incl. negative ones) of validation studies using non‐animal products and ingredients	Adequate funding; Expertise; Awareness / promoting validation of animal‐free products; expanding the use of animal‐free materials and reagents	Geneva Antibody Facility [53];
8	Allocate funding to research projects focused on replacement of animal‐derived products/ingredients with non‐animal alternatives	Awareness; Expertise / Increased availability of animal‐free alternatives; Increased use of animal‐free alternatives; increased expertise	Funding Bodies; NC3Rs [49]; PETA [76]; I‐Care [77]; OSA [32]

It would be also interesting to expand the current analysis by investigating the awareness of 3Rs centers in the various participants’ countries (e.g., to understand whether interactions with 3R centers occurred).

For the sake of completeness, it has also to be noted that this survey focussed only on reagents derived from animals covered by the scope of Directive 2010/63/EU. For example, reagents derived from arthropods (which are not covered by the Directive) are not part of this survey, neither are methods involving the use of whole animals. Nonetheless, reagents like Limulus Amebocyte Lysate (LAL), Tachypleus Amebocyte Lysate (TAL) as well as the Rabbit Pyrogen Test (RPT), an in vivo method which was already developed in 1912 [56], are used in laboratories worldwide to detect pyrogenic contaminations. LAL and TAL [57] are cell extracts from the hemolymph of different horseshoe crab species, at least two are already considered vulnerable [58] or even endangered [59] by the International Union for Conservation of Nature's Red List of Threatened Species, respectively. However, the European Pharmacopoeia included a synthetic substitute (recombinant Factor C, rFC) in 2016 [57] and a method containing reagents deriving from human blood (Monocyte‐Activation Test, MAT) in 2010 [60]. MAT and rFC will hopefully eliminate the need for harvesting horseshoe crab hemolymph and end the use of the RPT [61, 62]. Accordingly, a possible subsequent survey should also include reagents from animals outside of the scope of Directive 2010/63/EU and in vivo test methods, as these can affect reproducibility, reliability, translatability, and data consistency in between and within laboratories as well.

## 5 CONCLUDING REMARKS

This survey represents a first effort to map the current state of use of animal‐derived reagents across different sectors and to identify the hurdles possibly hampering the large implementation and use of non‐animal derived alternatives. The majority of the survey participants, mostly representing the academic environment, are aware of the potential issues associated with animal‐derived ingredients, especially ethical issues and batch‐to‐batch variability. The survey results also demonstrated that currently the main driver for the use of animal‐free alternatives is personal choice and that external pressures, such as from journals or funding bodies, are not promoting the use of alternatives. A high proportion of survey respondents identified issues with the availability or cost of animal free alternatives or reluctance towards the replacement of existing methodologies as the main reasons for not implementing non‐animal reagents. Moreover, the adequacy of information or education received on currently available animal‐free alternatives materials and reagents is generally scarce, as frequently declared by students and post‐docs. These conclusions formed the basis for the series of proposed initiatives (Table 3). Implementing these initiatives will bring not only ethical but also scientific benefits, considering that there is an urgent scientific need to replace animal‐derived, chemically‐undefined materials and reagents with chemically‐defined, non‐animal alternatives, especially in the light of today's research reproducibility crisis.

## CONFLICT OF INTEREST

None to declare.

## Supporting information



Supporting Information

Supporting Information

Supporting Information

## Data Availability

The data that support the findings of this study are available from the corresponding author upon reasonable request.
