# Supplementary material for: A worldwide survey on the use of animal‐derived materials and reagents in scientific experimentation
Source: Eng Life Sci. 2022 Jul 18;22(9):564–83. doi: 10.1002/elsc.202100167 (PMC9444711; doi:10.1002/elsc.202100167)
Supplement: Supplementary file 1 — Supporting Information [file ELSC-22-564-s003.docx]

**Supplementary information**

**A worldwide survey on the use of animal-derived materials and reagents in scientific experimentation**

*Manuela Cassotta^1^, Joanna Bartnicka^2^, Francesca Pistollato^2^, Surat Parvatam^3^, Tilo Weber^4^, Vito D’Alessandro^1^, Luisa Bastos^5^, and Sandra Coecke^2^*

^1^Oltre la Sperimentazione Animale (OSA), 20054 Segrate, Milan, Italy;

^2^European Commission, Joint Research Centre (JRC), Ispra, Italy;

^3^Centre for Predictive Human Model Systems, Atal Incubation Centre-Centre for Cellular and Molecular Biology (AIC-CCMB), Hyderabad 500 039, India;

^4^Animal Welfare Academy of the German Animal Welfare Federation, Neubiberg, Germany; ^5^Eurogroup for animals

**Survey questions**

**Personal information**

1. What kind of organisation do you work for?

- Pharmaceutical company
- Biotechnology company
- Cosmetics company
- Academia/Academic institution
- Regulatory body
- Governmental institution
- Non-profit/non-governmental institution
- Educational institution (e.g., high school, etc.)
- Other

2. Please select the option that best describes your role within your organisation.

- Student/PhD student
- Lab technician
- Research assistant
- Post-doctoral researcher/Research fellow
- Senior scientist/Team leader
- Department head
- Professor/Teacher/Lecturer
- Senior manager
- Science communicator
- Lobbyist
- Policy/decision-maker
- Other

3. How would you rate your level of responsibility or decision-making power within your organisation?

- High
- Medium
- Low

4. Geographical position (Select Country)

**Methodological approaches, materials and reagents**

5. What is your main field of research or professional activity? (You may select more than one option)

- Basic/fundamental research
- Translational/applied research
- Clinical research
- Epidemiological research
- Regulatory/policy/decision-making
- Education
- Communication/marketing

6. What methodological approach(es) do you use most or are you most familiar with in your research or professional activity? at most 3 choice(s)

- In vivo/animal models
- Ex-vivo
- In vitro/human or animal cell-based or cell-free models
- In silico/computational models
- Creation of educational/dissemination material
- Material design/marketing
- Other

7. What kind of in vitro test systems do you use most or are you most familiar with in your research or professional activity? at most 3 choice(s)

- In chemico
- Whole animal or human cell-derived materials (e.g., proteins/enzymes, sub-cellular fractions, DNA, RNA)
- Human continuous/finite cell lines
- Animal continuous/finite cell lines
- Human primary cell cultures/dissociated cells
- Animal primary cell cultures/dissociated cells
- Human stem cells and/or their differentiated derivatives
- Animal stem cells and/or their differentiated derivatives
- Human tissues/organ explants
- Animal tissues/organ explants
- Complex culture systems
- Other
- Not applicable

8. How much time and resources do you dedicate to in vitro experimentation in your research or professional activity?

- High (most of my time/resources)
- Medium (part of my time/resources)
- Low (limited time/resources)
- Not applicable

9. What types of animal-derived materials and reagents do you use most or are you most familiar with in your research or activities? (You may select more than one option)

- Serum
- Growth factors
- Dissociation enzymes (e.g., trypsin, collagenase, papain, nucleases, etc.)
- Coating materials (e.g., laminin, Matrigel™, etc.)
- Antibodies (e.g., monoclonal, polyclonal)
- Other
- Not applicable

**Awareness**

10. In your view, what are the main issues associated with the use of animal-derived materials and reagents? (You may select more than one option)

- Batch to batch variations/Low reproducibility of scientific results
- Interspecies differences/Low translatability to humans
- Risk of material cross-contamination
- Risk of pathogen contamination (e.g., virus, bacteria, fungi, etc.)
- Presence of undefined/unknown components
- Ethical concerns
- Other
- I am not aware of any issues associated with animal-derived reagents

11. Have you ever considered the use of animal-free alternatives for any of the animal-derived materials and reagents you normally work on or are familiar with in your research or professional activity? (You may select more than one option)

- Yes, alternatives to animal-derived serum
- Yes, alternatives to animal-derived growth factors
- Yes, alternatives to animal-derived dissociation enzymes
- Yes, alternatives to animal-derived coating materials
- Yes, alternatives to animal-derived antibodies
- Yes, alternatives to other animal-derived material or reagent
- No
- Not applicable

12. What were/are the main factors that made you consider such animal-free alternative(s)? (You may select more than one option)

- Requested from superiors
- Stimulated by colleagues
- Grant/funding application rules/recommendations
- Animal-free ingredients were/are already included in protocols developed, used or validated in my lab/institution
- Commercial availability
- National 3R centre recommendations
- OECD Good In vitro Method Practice Guidance document (GIVIMP)
- Incentives via scientific publications, scientific journal policies
- My personal choice
- Other
- Not applicable

13. What were/are the main reasons for not considering animal-free alternatives? (You may select more than one option)

- I was/am not aware of their availability
- I did/do not know where to purchase them
- I prefer(red) to stick to protocols already developed/used/validated in my lab/institution
- I was/am worried about the cost or not having funds
- I had/have no decision-making power on this matter
- I did/do not have enough knowledge to decide on this matter
- I thought/think animal-derived materials/reagents are not replaceable
- Other
- Not applicable

14. Do you think that animal-free alternative materials and reagents offer any benefit over animal-derived materials and reagents? (You may select more than one option)

- Ethical benefits (e.g., reduce or avoid animal sufferance)
- Biosafety benefits
- Scientific relevance/reliability benefits
- Reproducibility benefits
- Economic/cost benefits
- I do not think that animal-free alternatives offer any benefit over animal-derived materials and methods
- I don't know/I never thought about that

15. In your view, what are the animal-derived materials and reagents whose production raises the highest ethical concern with regard to animal sufferance? (While selecting, please assign a score from 1 to 5, considering that 1 means ‘minimal level of sufferance’ and 5 ‘high level of sufferance’)

- Serum (1 2 3 4 5 Not Sure)
- Growth factors (1 2 3 4 5 Not Sure)
- Dissociation enzymes (1 2 3 4 5 Not Sure)
- Coating materials (1 2 3 4 5 Not Sure)
- Antibodies (1 2 3 4 5 Not Sure)
- Other material/reagent (1 2 3 4 5 Not Sure)

**Knowledge and information sources**

16. How would you rate your levels of awareness or knowledge about currently available animal-free alternative materials and reagents for in vitro experimentation?

- High
- Medium
- Low
- Extremely low/null
- Not sure

17. How do you judge the level of information on animal-free alternative materials and reagents received during your academic and/or professional experience?

- Adequate
- Sufficient
- Inadequate
- Not sure

18. In your view, what type of educational and dissemination sources could be useful or most impactful to learn more about animal-free alternative materials and reagents? at most 3 choice(s)

- Online seminars
- Online videos on demand
- Specific academic curricula
- Literature reviews/publications
- Websites/web platforms
- Ad hoc meetings (e.g., conferences, summer schools, workshops, etc.)
- Specialization/professional coursesDedicated social media accounts
- Journal GIVIMP compliance policies
- Other

19. Would you like to know more about animal-free alternative materials and reagents?

- Yes (please, indicate your email address if you wish to be contacted)
- No, not interested
