# Supplementary material for: A worldwide survey on the use of animal‐derived materials and reagents in scientific experimentation
Source: Eng Life Sci. 2022 Jul 18;22(9):564–83. doi: 10.1002/elsc.202100167 (PMC9444711; doi:10.1002/elsc.202100167)
Supplement: Supplementary file 2 — Supporting Information [file ELSC-22-564-s001.docx]

**Supplementary information**

**A worldwide survey on the use of animal-derived materials and reagents in scientific experimentation**

*Manuela Cassotta^1^, Joanna Bartnicka^2^, Francesca Pistollato^2^, Surat Parvatam^3^, Tilo Weber^4^, Vito D’Alessandro^1^, Luisa Bastos^5^, and Sandra Coecke^2^*

^1^Oltre la Sperimentazione Animale (OSA), 20054 Segrate, Milan, Italy;

^2^European Commission, Joint Research Centre (JRC), Ispra, Italy;

^3^Centre for Predictive Human Model Systems, Atal Incubation Centre-Centre for Cellular and Molecular Biology (AIC-CCMB), Hyderabad 500 039, India;

^4^Animal Welfare Academy of the German Animal Welfare Federation, Neubiberg, Germany; ^5^Eurogroup for animals

**Supplementary information about applied search terms in PubMed to generate targeted email lists**

Search was conducted using PubMed (in March-May 2021) applying the list of keywords (with MeSh terms) and Boolean operators indicated in the table below.

| **Target audience** | **Keywords combinations** |
| --- | --- |
| “In vivo” scientists | (((animal) AND (model)) OR (models)) OR (in vivo) |
| “In vitro” scientists | (("fetal bovine serum") OR ("fetal calf serum")) AND (cell culture[MeSH Terms]) (growth factor) AND (cell culture)  ((((in vitro) OR (organ-on-a-chip)) OR (cell-culture)) OR (organoid[MeSH Terms])) OR (organoids) |
| Affiliated to Pharmaceutical / Biotechnological companies | ((((((((((((((((((((Novo Nordisk[Affiliation]) OR (Regeneron Pharmaceuticals[Affiliation])) OR (Vertex[Affiliation])) OR (Gilead[Affiliation])) OR (Alexion[Affiliation])) OR (Incyte[Affiliation])) OR (biomarinpharmaceuticals[Affiliation])) OR (United Therapeutics[Affiliation])) OR (alkenes plc[Affiliation])) OR (Ionis Pharmaceuticals[Affiliation])) OR (aciesbio[Affiliation])) OR (Abbott[Affiliation])) OR (GlaxoSmithKline[Affiliation])) OR (Biogen[Affiliation])) OR (Seagen[Affiliation])) OR (Genmab[Affiliation])) OR (Bausch Health[Affiliation])) OR (Moderna[Affiliation])) OR (Mylan[Affiliation])) OR (alnilam pharmaceuticals[Affiliation])) OR (VRTX[Affiliation])  ((((((((((((((((JSK[Affiliation]) OR (Pfizer[Affiliation])) OR (Roche[Affiliation])) OR (Astrazeneca[Affiliation])) OR (Johnson&Johnson[Affiliation])) OR (Menarini[Affiliation])) OR (Sanofi[Affiliation])) OR (Merck[Affiliation])) OR (Novartis[Affiliation])) OR (Bayer[Affiliation])) OR (Eli Lilly[Affiliation])) OR (Abbvie[Affiliation])) OR (Janssen[Affiliation])) OR (Alfasigma[Affiliation])) OR (Chiesi[Affiliation])) OR (Angelini[Affiliation])) OR (Recordati[Affiliation]) |
| Affiliated to Cosmetic companies | (((((((((Shiseido[Affiliation]) OR (Dove[Affiliation])) OR (Gillette[Affiliation])) OR (Nivea[Affiliation])) OR (panten[Affiliation])) OR (Garnier[Affiliation])) OR (Guerlain[Affiliation])) OR (Clinique[Affiliation])) OR (Estee Lauder[Affiliation])) OR (L'Oreal[Affiliation]) |
| “In silico” scientists | ((in silico) OR (computational)) AND (model) |
