# Supplementary material for: A worldwide survey on the use of animal‐derived materials and reagents in scientific experimentation
Source: Eng Life Sci. 2022 Jul 18;22(9):564–83. doi: 10.1002/elsc.202100167 (PMC9444711; doi:10.1002/elsc.202100167)

Cassotta M. et al. A worldwide survey on the use of animal-derived materials and reagents in scientific experimentation

Supplementary Material

Disaggregation by the type of organisation

Number of respondents per organisation type were as follows:

| Academia/Academic institution :342 |
| --- |
| Biotechnology company : 35 |
| Cosmetics company : 5 |
| Educational institution (e.g., high school, etc.): 44 |
| Governmental institution : 43 |
| Non-profit/non-governmental institution : 34 |
| Other : 30 |
| Pharmaceutical company : 13 |
| Regulatory body : 5 |

Disaggregated analysis of survey answers given by 5 biggest subgroups (Academia, Biotechnology companies, Educational institutions, Governmental institutions, Non-profit/non-governmental institutions) are presented below. Pharmaceutical companies, cosmetics companies and regulatory bodies were not analysed separately in this analysis due to low number of replies, however, pooled replies from biotechnology, pharmaceutical and cosmetics companies were considered as a category ‘Industry’.

Supplementary Figure 1: Relationship between the type of organisation and considering the use of animal-free alternatives.

*A*mong all types of organisations, biotechnology companies had the highest percentage of respondents, who considered to use at least one animal-free alternative of animal-derived ingredients that they normally work on (77%, compared with the average of 60%).


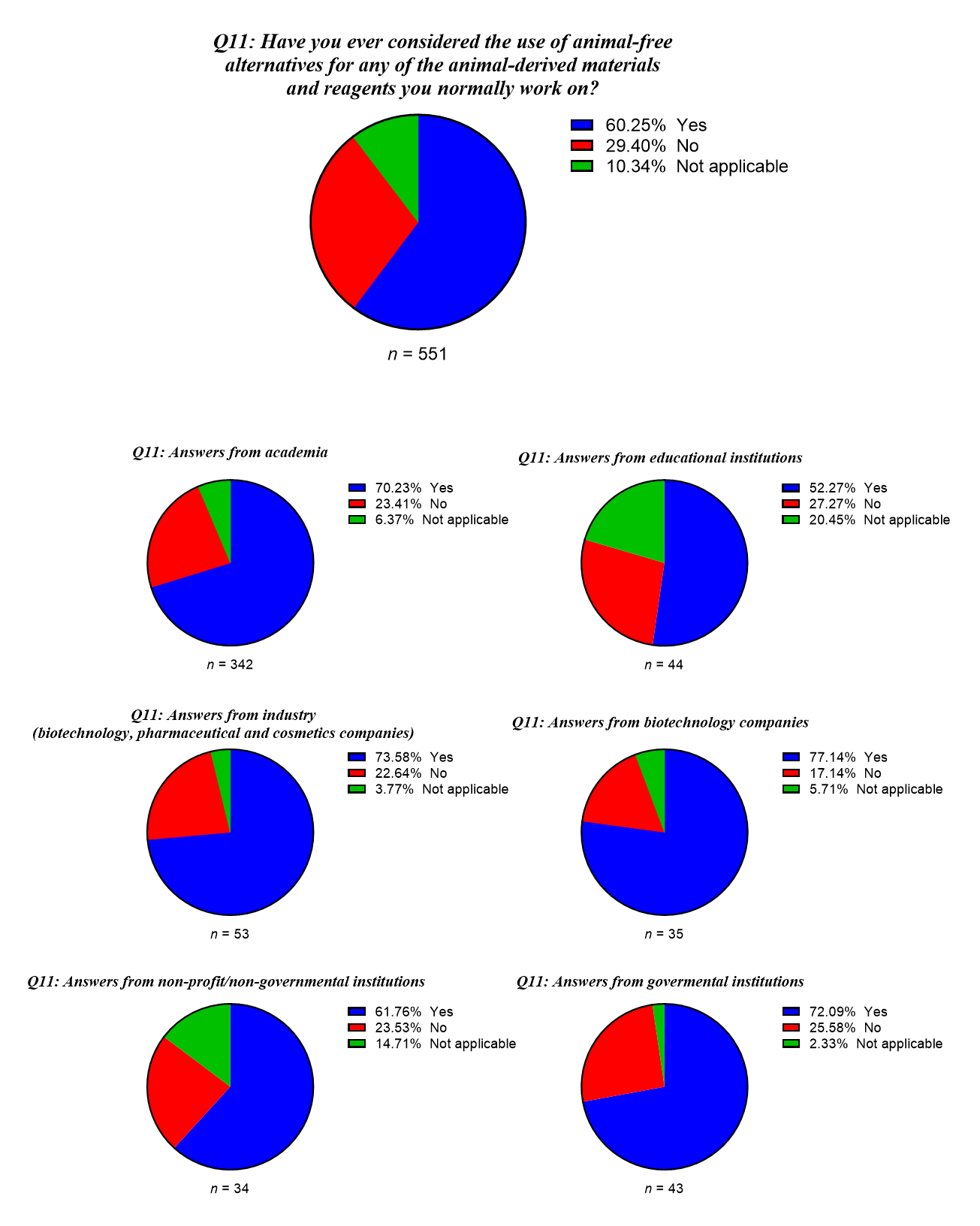


Supplementary Figure 2: Relationship between the type of organisation and level of knowledge/awareness on the animal-free alternatives


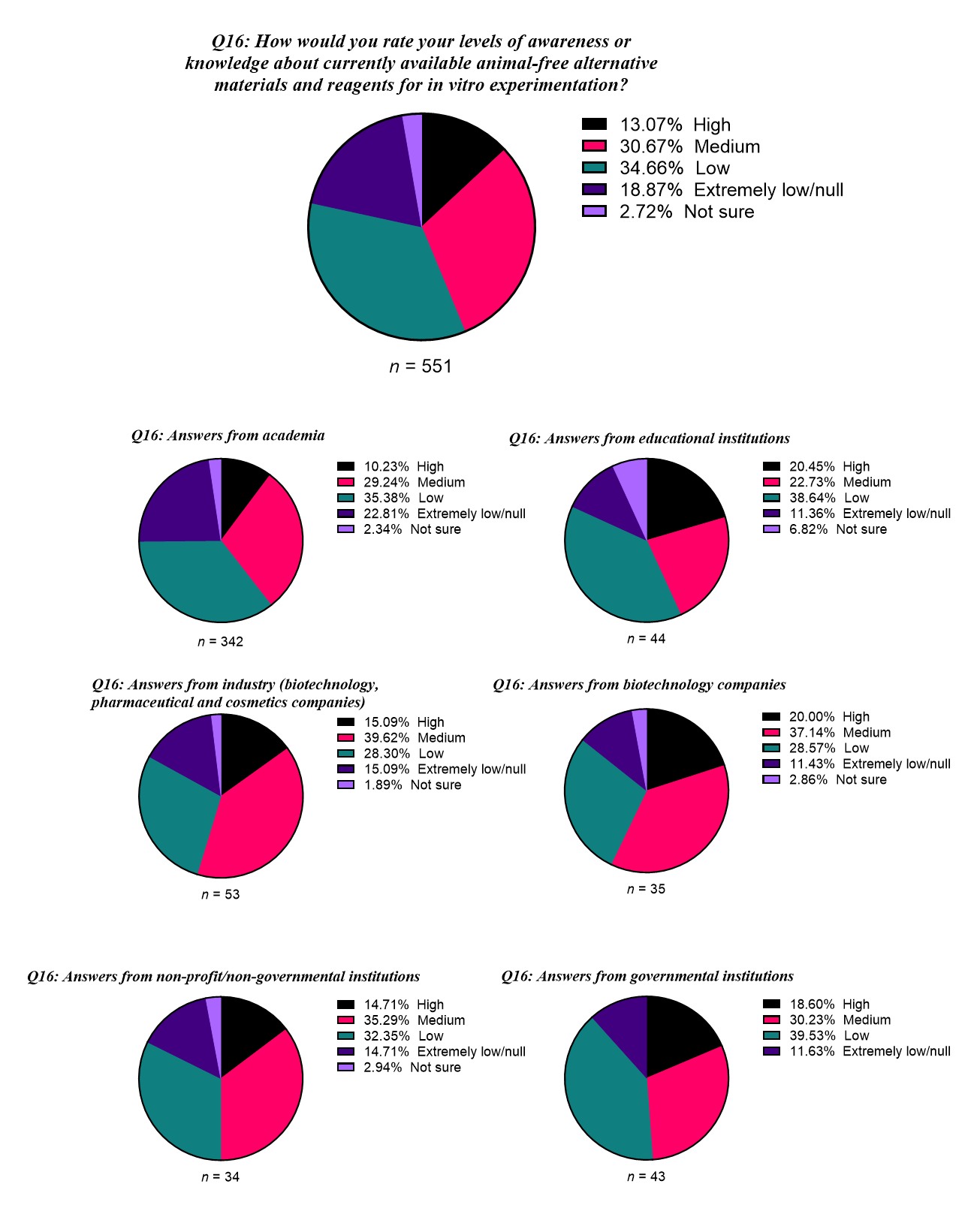
Among all types of organisations, biotechnology companies rated the highest their awareness or knowledge on the currently available animal-free materials (20% rated as high and 37% as medium, compared with the pooled respondents, out of which 13% rated high and 31% medium). Among respondents in academia, only 10% rated their awareness/knowledge as high and 29% as medium.

Supplementary Figure 3: Relationship between the type of organisation and level of information received on the animal-free alternatives during academic and professional experience

**
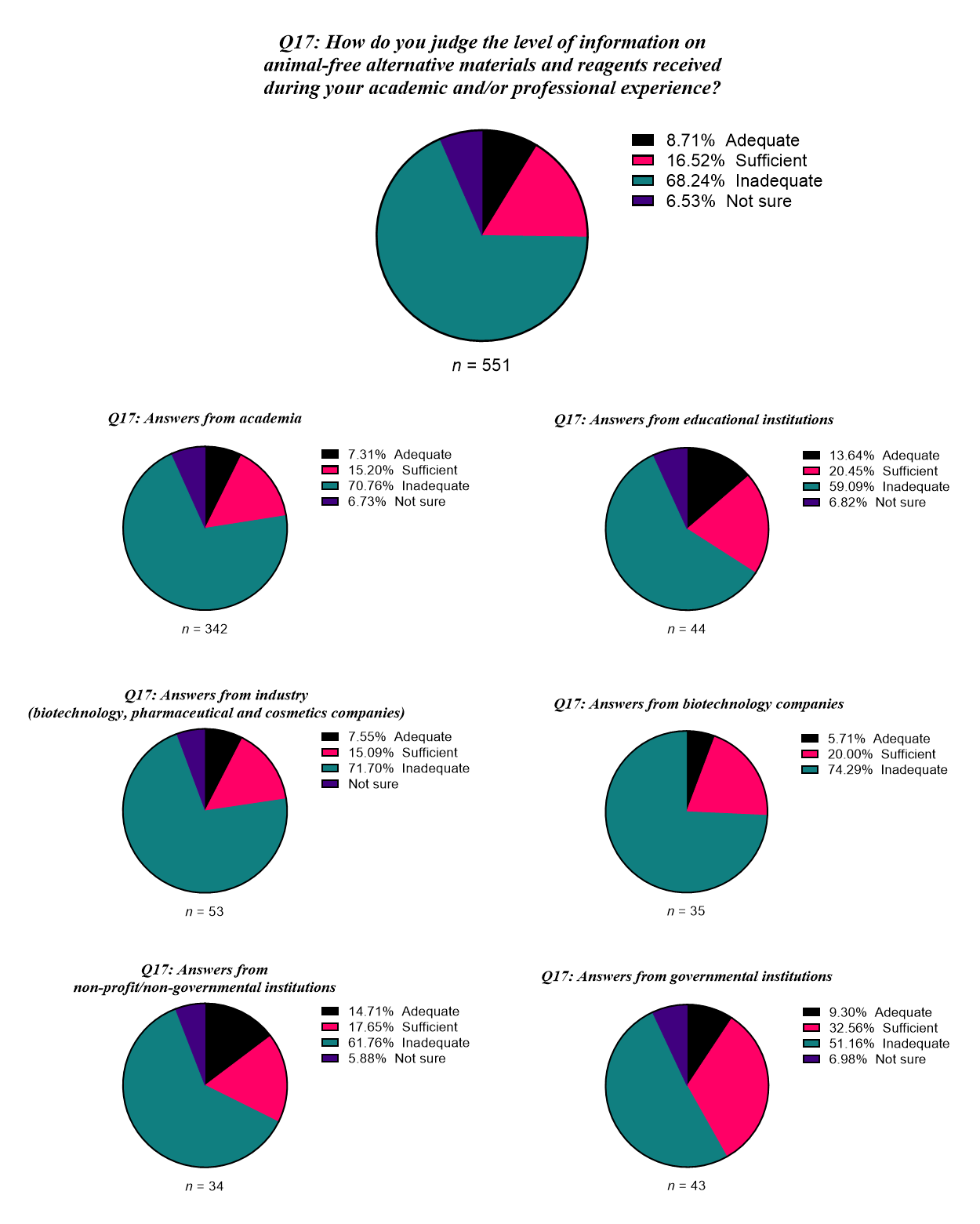
**Among all types of organisations, governmental institutions rated the highest the level of information received on the animal-free alternatives (9% rated as adequate and 33% as sufficient, compared with the pooled respondents, out of which 9% rated adequate and 17% sufficient). It is notable that in all types of organisations, vast majority of respondents deemed it as inadequate.

Disaggregation by the role/job title

Number of respondents per role/job title were as follows:

| Department head : 30 |
| --- |
| Lab technician : 13 |
| Lobbyist : 4 |
| Other : 14 |
| Policy/decision-maker : 2 |
| Post-doctoral researcher/Research fellow: 70 |
| Professor/Teacher/Lecturer :136 |
| Research assistant : 11 |
| Science communicator : 7 |
| Senior manager : 16 |
| Senior scientist/Team leader :137 |
| Student/PhD student :111 |

Disaggregated analysis of survey answers given by the 4 biggest subgroups (Post-doctoral researcher/Research fellow, Professor/Teacher/Lecturer, Senior scientist/Team leader and Student/PhD student) are presented below.

Supplementary Figure 4: Relationship between the role within one’s organisation and considering the use of alternatives

Smaller percentage of students considered the use of any animal-free alternatives, compared to all other groups.


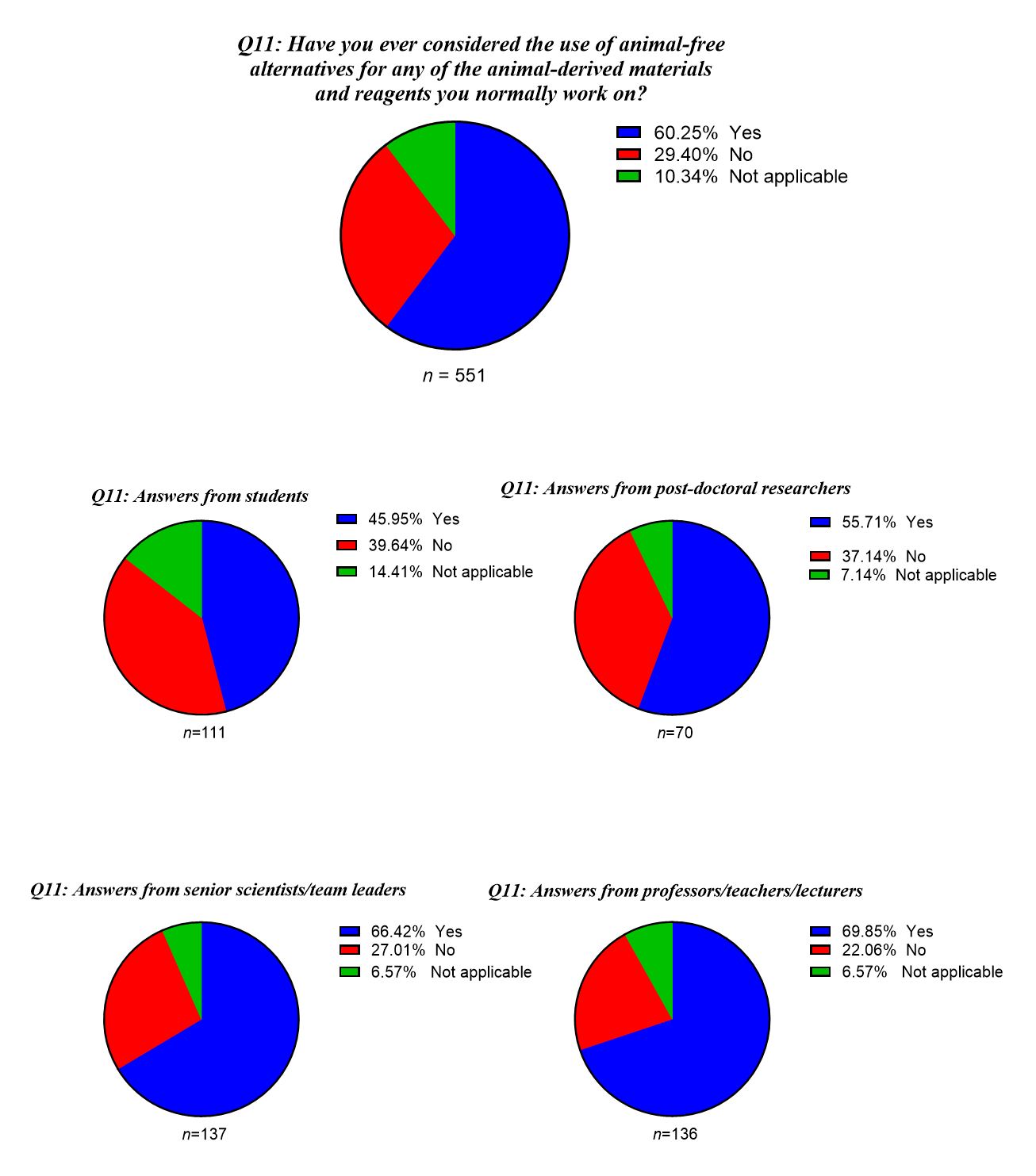


Supplementary Figure 4: Relationship between the role within one’s organisation and level of knowledge/awareness on the animal-free alternatives


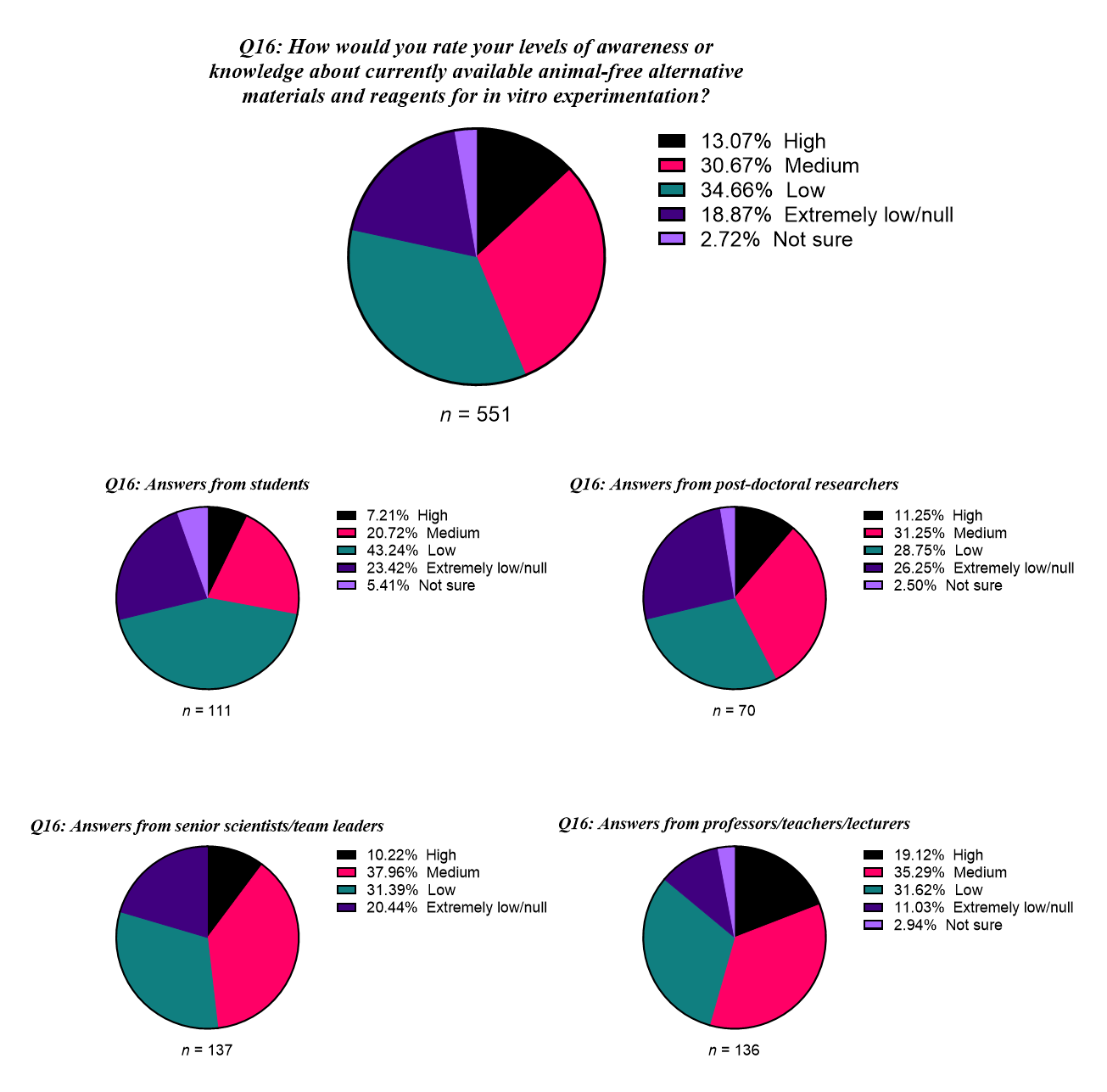
Students less frequently rated their level of awareness on animal-free alternatives as high (7%) or medium (21%) compared to post-docs (11% high, 31 % medium), senior scientists (10%, 38% medium), teachers, professors or lecturers (19% high, 35% medium).

Supplementary Figure 5: Relationship between the role within one’s organisation and level of information received on the animal-free alternatives


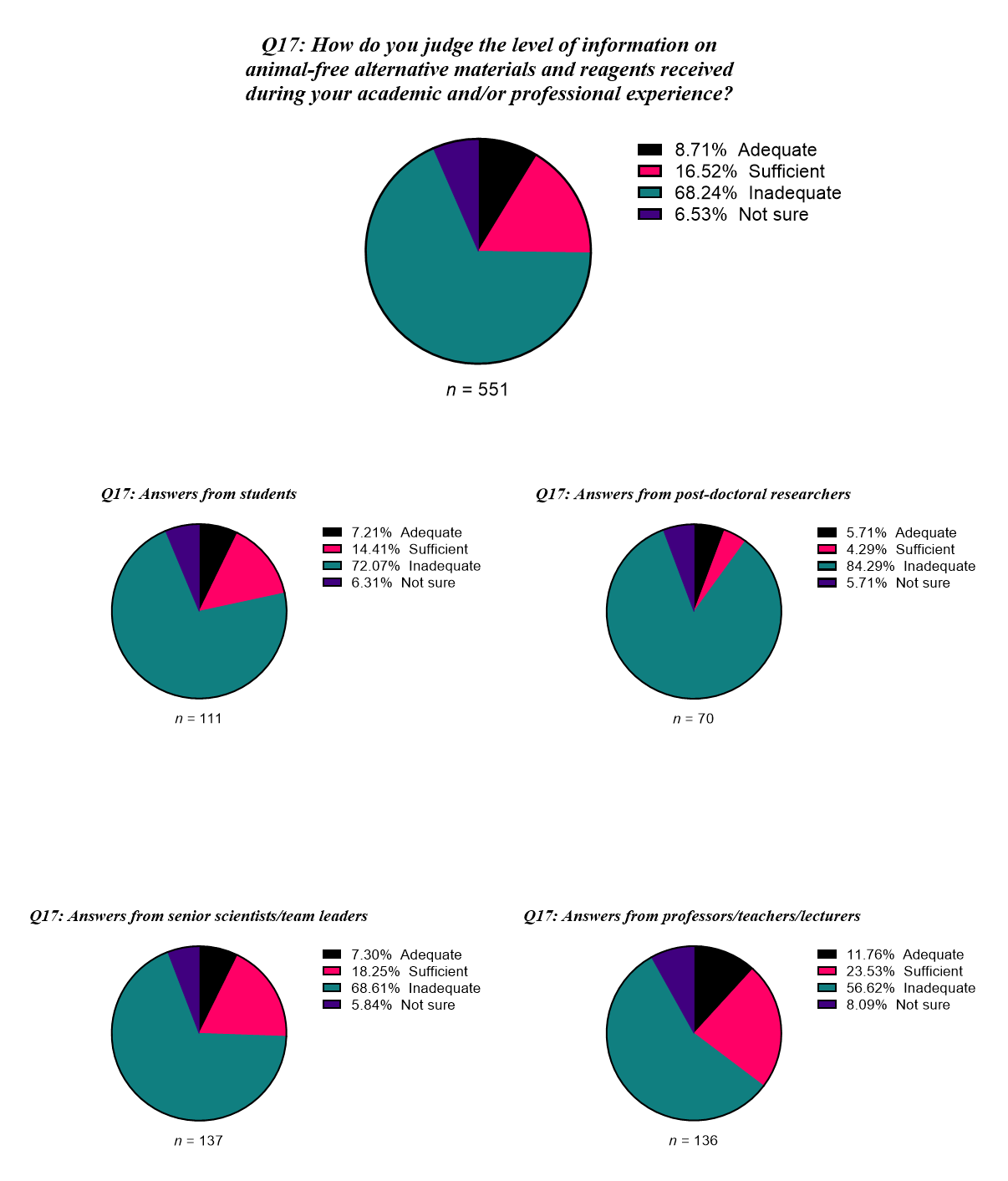
Higher proportion of students and post docs rated the level of education/information on alternatives that they received as inadequate (72% of students, 84% of post-docs), compared to senior scientists (68%) , teachers, professors or lecturers (57%).

Supplementary Figure 6: Relationship between the role within one’s organisation and willingness to know more about alternatives

Higher proportion of students (69%) and post-docs (69%) demonstrated the willingness to know more about the animal-free alternatives, compared to senior scientists (55%), teachers, professors or lecturers (57%).


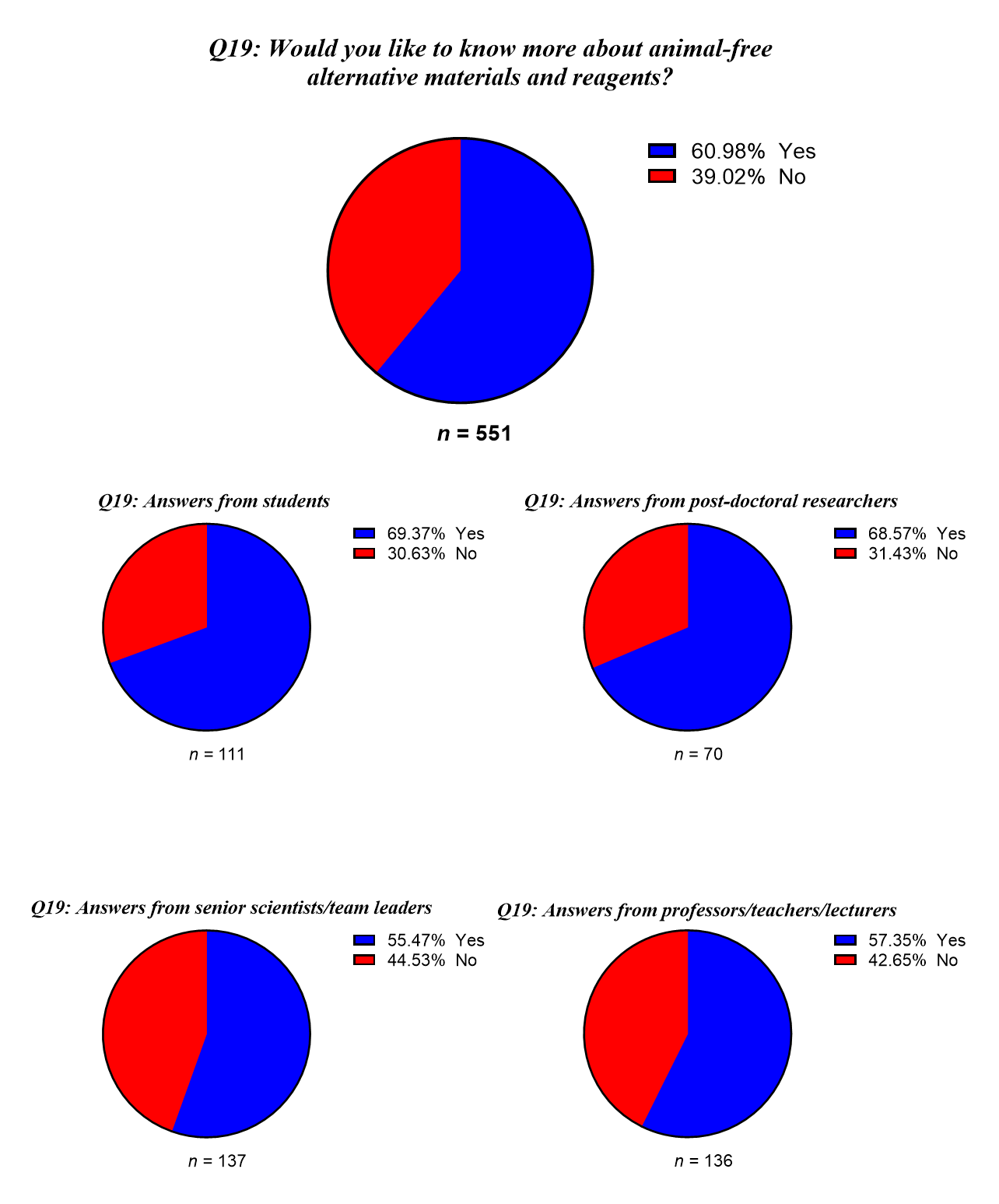


Disaggregation by the type of research

In the first subgroup analysis, answers were filtered to retrieve respondents who either only worked in basic/fundamental research (136) or only in the translational/applied research (96). The comparison of groups who chose at least one of these options was not performed because 174 respondents selected both basic and translational research and therefore, there was a large overlap between the groups. Since only 17 respondents chose as their only option clinical research, this subgroup was not separately analysed due to small sample size.

In the second analysis, answers were filtered to retrieve respondents who declared to work with ‘human stem cells and/or their differentiated derivatives’ or ‘animal stem cells and/or their differentiated derivatives’. 116 respondents selected at least one of these options.

Supplementary Figure 7: Consideration of animal-free alternatives among basic/fundamental and translational/applied research scientists

Respondents dedicated to basic/fundamental research had a similar pattern of reply to these dedicated to translational/applied research, when asked if they have considered animal-free alternatives to animal-derived materials they used.

Supplementary Figure 8: Level of awareness or knowledge about animal-free alternatives among basic/fundamental and translational/applied research scientists

Compared to the respondents dedicated to basic/fundamental research, these dedicated to translational/applied research more frequently rated their level of awareness or knowledge on animal-free alternatives as high (15% translational researchers vs 10% basic researchers) or medium (34% of translational researchers vs 27% of basic researchers).

Supplementary Figure 9: Level of information received about animal-free alternatives among basic/fundamental and translational/applied research scientists

Compared to the respondents dedicated to basic/fundamental research, these dedicated to translational/applied research rated only slightly more frequently the level of information they received on animal-free alternatives as adequate (9% translational researchers vs 7% basic researchers) or sufficient (17% of translational researchers vs 13% of basic researchers).


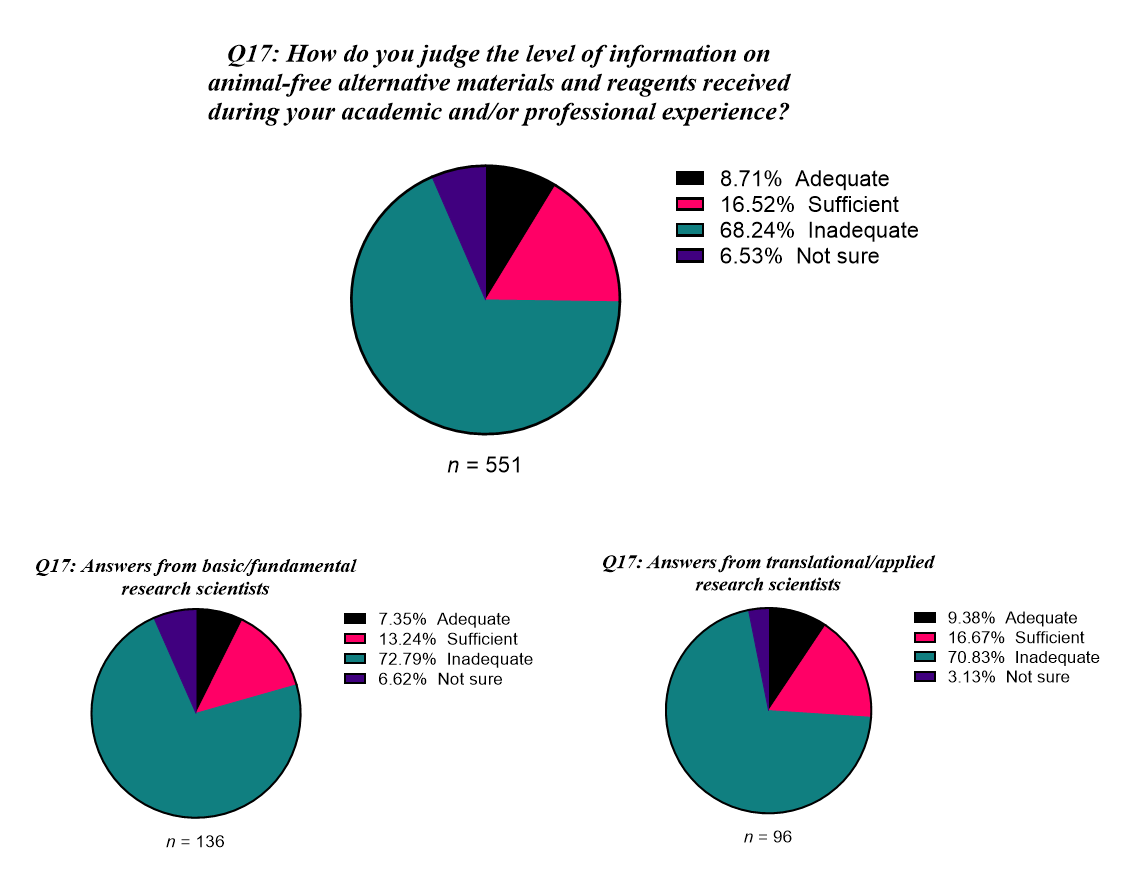


Supplementary Figure 10: Consideration of animal-free alternatives among stem cell scientist

Compared to all pooled respondents, higher proportion of stem cells scientists considered the use of at least one animal-free alternative (83% of stem cell scientists, compared to 60% of all pooled respondents).


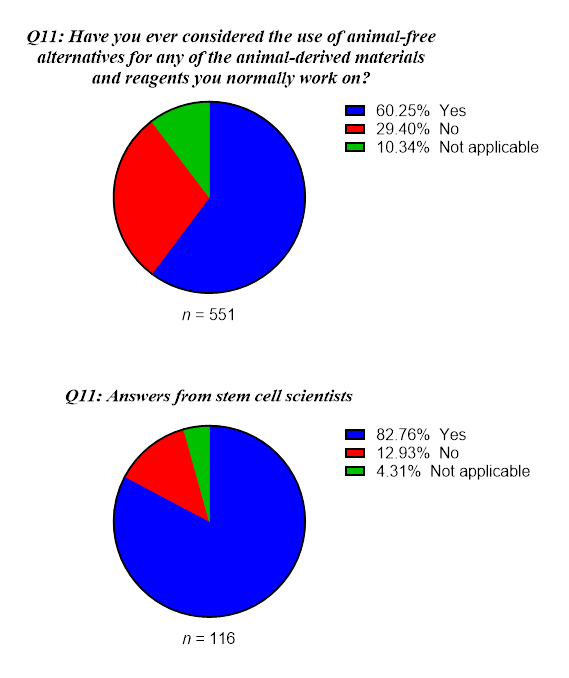


Supplementary Figure 11: Level of awareness or knowledge about animal-free alternatives among stem cell scientists

Compared to all pooled respondents, higher proportion of stem cells scientists rated the awareness/knowledge on animal-free alternatives as high (22% vs 13% of all respondents).


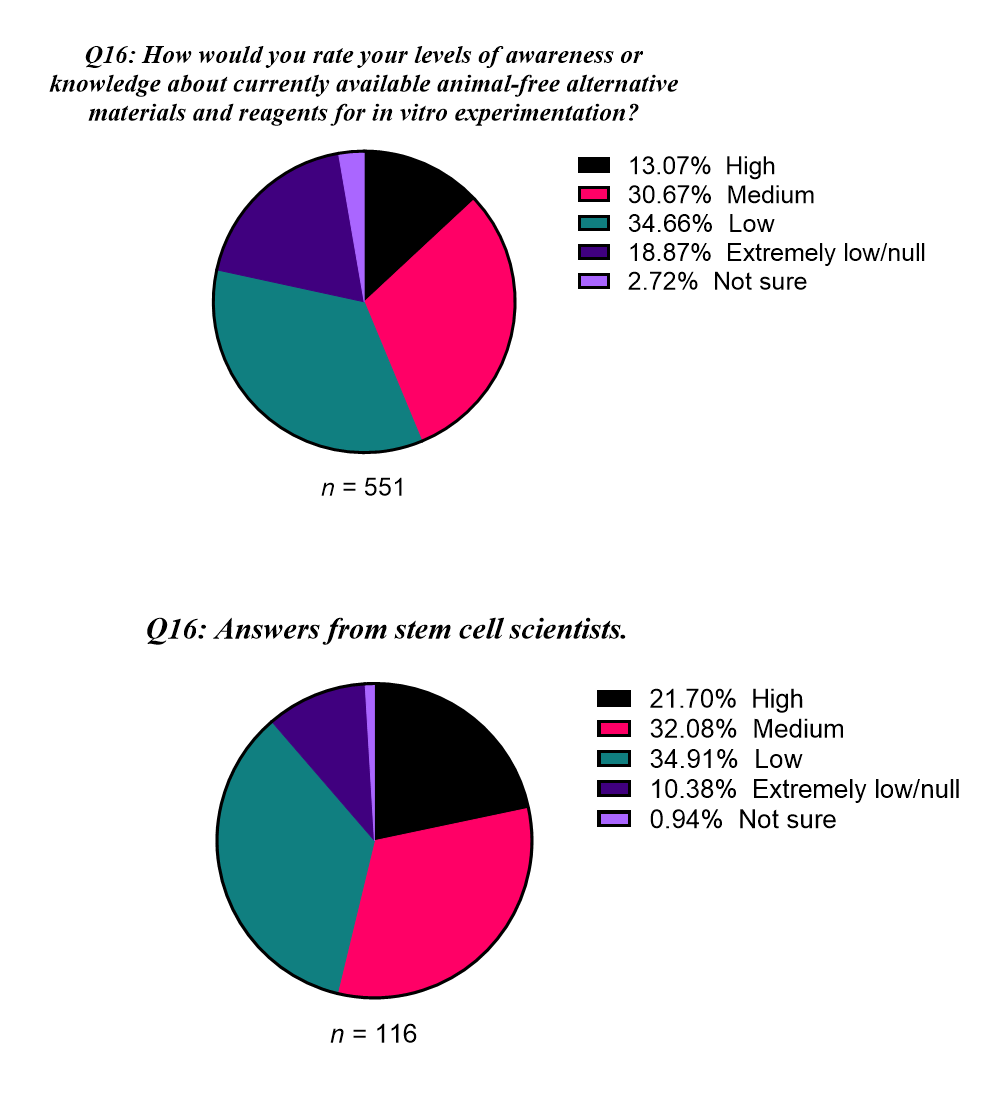


Supplementary Figure 12: Information on the animal-free alternatives received by stem cell scientists

Compared to all pooled respondents, slightly higher proportion of stem cells scientists rated the information they received on animal-free alternatives as adequate (13% vs 9% all respondents) or sufficient (19% vs 17% all respondents).

Analysis of a subgroup of respondents who did not perceive any issues with animal-derived reagents or have not considered the alternatives

The following analysis focuses on the subsets respondents, who indicated that they do not perceive any issues with animal-derived ingredients (Q10, 45 respondents gave that answer)) or that they have never considered the use of alternatives (Q11, 162 respondents gave that answer).

Supplementary Figure 13: Relationship between perceived lack of issues with animal-derived reagents or not considering alternatives and level of knowledge/awareness on the animal-free alternatives

*Compared to the average respondent, these who were not aware of the issues with animal-derived ingredients or have never considered their use, more frequently also indicated lower levels of awareness on the animal-free alternatives.*


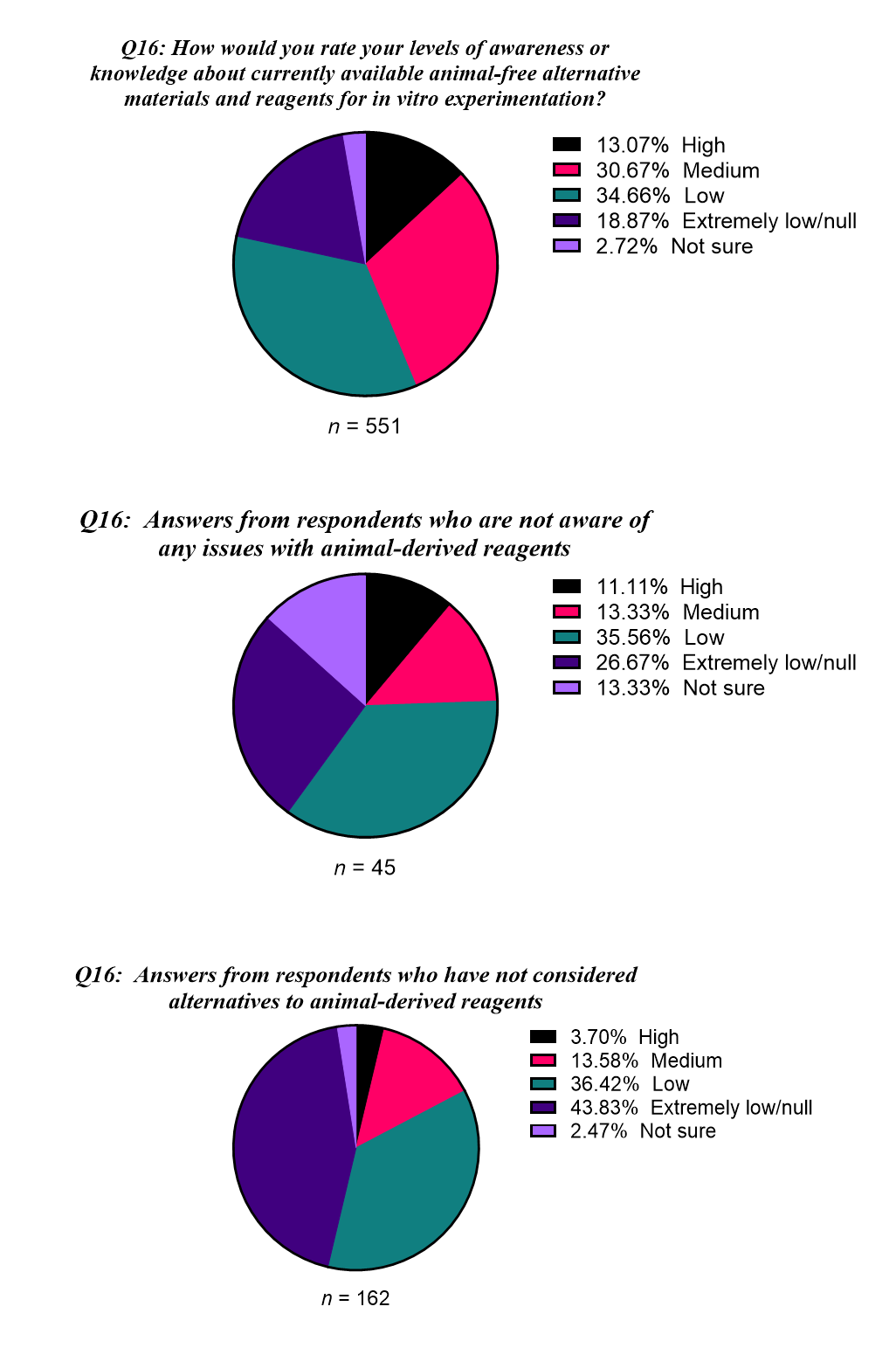


Supplementary Figure 14: Relationship between perceived lack of issues with animal-derived reagents or not considering alternatives and level of information received on the animal-free alternatives

*Compared to the average respondent, these who never considered the use of alternatives more frequently also indicated inadequate level of knowledge received, while these who were unaware of the issues were more frequently unsure whether their knowledge was adequate.*


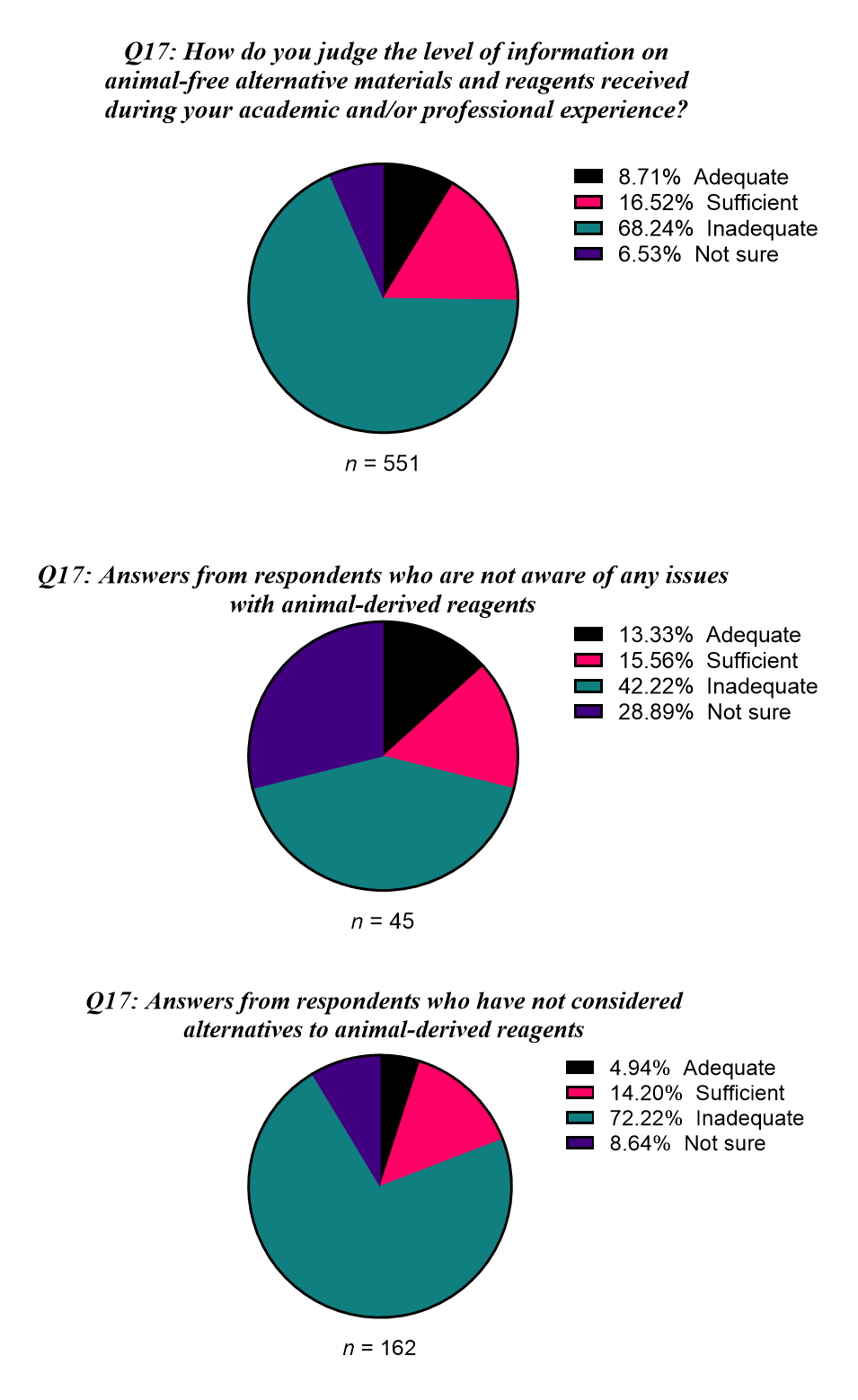


Supplementary Figure 15: Relationship between perceived lack of issues with animal-derived reagents or not considering alternatives and the willingness to know more

*Respondents who have never considered the use of alternatives indicated in majority expressed the willingness to be contacted to learn more (60%), similarly to the pooled respondents (61%), while among the respondents not aware of any issues with animal-derived reagents, the proportion of these who are willing to learn more was much less (31%)*


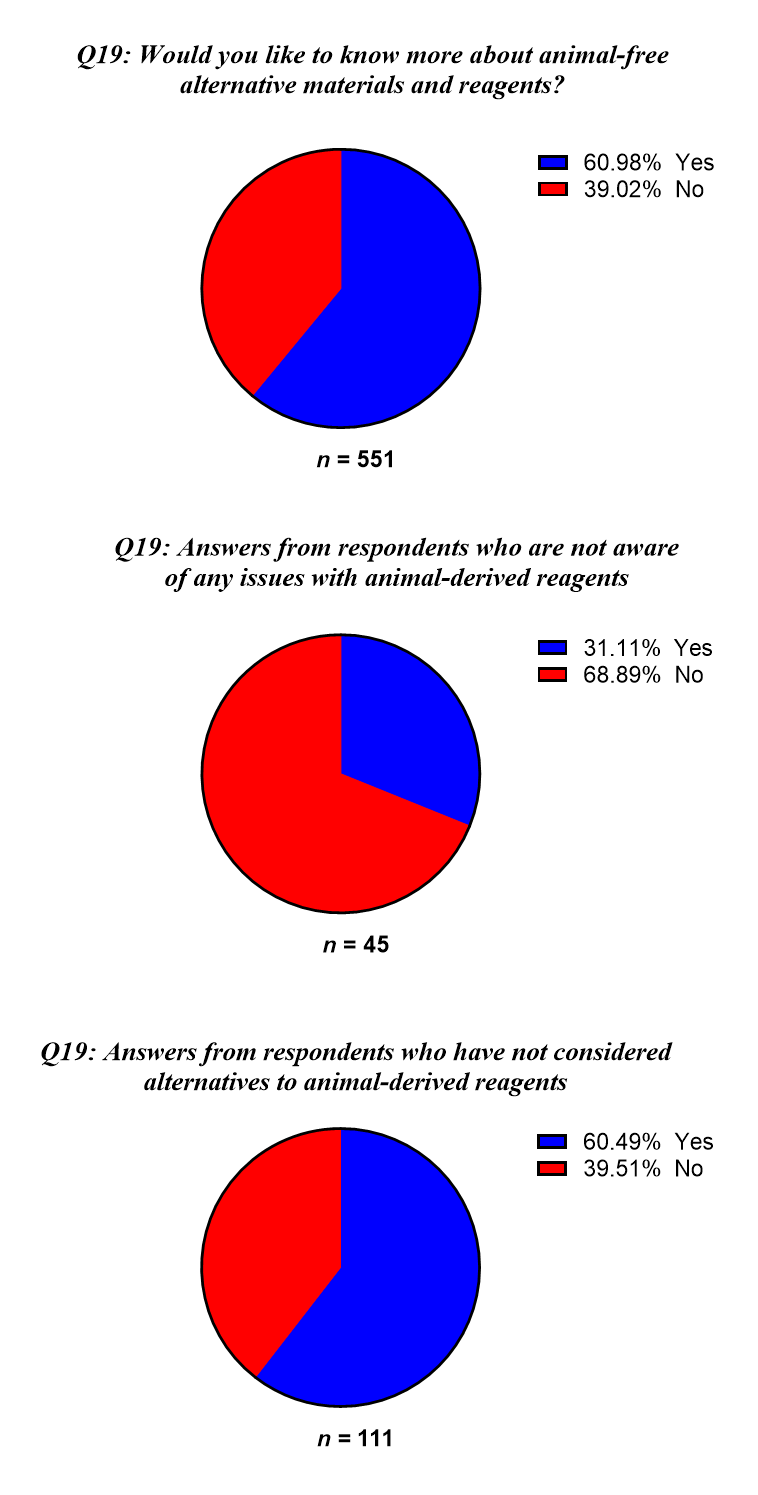

Supplement: Supplementary file 3 — Supporting Information [file ELSC-22-564-s002.docx]
